# Effect of aromatherapy on quality of life in maintenance hemodialysis patients: a systematic review and meta-analysis

**DOI:** 10.1080/0886022X.2022.2164202

**Published:** 2023-03-12

**Authors:** Cong Zhang, Hang Mu, Yong-Fang Yang, Yong Zhang, Wen-Jun Gou

**Affiliations:** a Department of Cardiothoracic and Vascular Surgery, The First Affiliated Hospital of Yangtze University, Jingzhou, PR China; b Department of Oncology, Jianli People’s Hospital, Jianli, PR China; c Department of Nursing, Jianli People’s Hospital, Jianli, PR China; d Department of Nephrology, Jianli People’s Hospital, Jianli, PR China; e Department of Nephrology, The First Affiliated Hospital of Yangtze University, Jingzhou, PR China

**Keywords:** Aromatherapy, meta-analysis, essential oils, inhalation, hemodialysis, restless legs syndrome, fatigue, anxiety, sleep quality, randomized controlled trials, integrative medicine

## Abstract

**Objective:** Aromatherapy has been used for patients on maintenance hemodialysis (MHD), but the outcomes are still controversial. Thus, we conducted this study to systematically evaluate the effect of aromatherapy on the quality of life of patients on MHD.

**Methods:** We searched the PubMed, Embays, Scopus, Web of Science, and CNKI databases for randomized controlled trials that evaluated the use of aromatherapy in dialysis patients and reported at least one outcome of interest.

**Results:** Twenty-two relevant studies were included in the meta-analysis. The meta-analysis revealed that aromatherapy significantly increased subjective sleep quality (a lower score indicates better sleep quality) [standardized mean difference (SMD) = −1.52, 95% CI (−2.38, −0.67), *p* < 0.01] and reduced fatigue [SMD = −1.14, 95% CI (−1.95, −0.33), *p* = 0.01], anxiety [SMD = −1.38, 95% CI (−2.09, −0.67), *p* < 0.01], symptoms of restless legs syndrome [RLS; SMD = −1.71, 95% CI (−2.09, −1.33), *p* < 0.01], and arteriovenous fistula puncture pain [SMD= −1.56, 95% CI (−2.60, −0.52), *p* < 0.01].

**Conclusions:** Aromatherapy may be used as a novel complementary and alternative therapy to improve sleep quality and reduce fatigue, anxiety, symptoms of RLS, and arteriovenous fistula puncture pain in patients on MHD.

## Introduction

End-stage renal disease (ESRD), also known as uremia, is the terminal stage of various chronic kidney diseases (CKDs). ESRD has become a major human health threat worldwide [1]. The findings of Lonazno et al. demonstrated that 100–2500 people per million suffer from ESRD worldwide, which is currently ranked as the 18th leading cause of death globally [2]. Although dialysis technology is advancing, leading to improvements in patient survival, most patients in the process of treatment also experience ESRD, and the treatment of associated symptoms, including fatigue, disordered sleep, arteriovenous fistula puncture pain, anxiety, and pressure, are associated with poor health-related quality of life (HRQoL) [3–6]. Although medications (such as l-carnitine, growth hormone, nandrolone decanoate, and vitamin C supplementation) are effective in managing such symptoms, prolonged use may lead to drug dependence or long-term exacerbation of symptoms [7,8]. In recent years, the use of complementary and alternative medicine (CAM) to improve patient health has become increasingly popular [9]. Acupressure, reflexology, and massage therapies are the most well-studied manipulative and body-based methods (MBMs) that have demonstrated efficacy in alleviating sleep disturbance, fatigue, and uremic pruritus (UP) symptoms in CKD patients [10].

Aromatherapy is the use of concentrated essential oils extracted from herbs, flowers, and other plant parts to treat various diseases [11]. The proponents of aromatherapy lay claim to an ancient tradition of herbal medicine practiced in countries, such as Egypt and India thousands of years ago. However, the term was initially used by the French chemist Gattefossé in a book first published in 1936 [12]. These oils are now commonly administered by massaging them into the skin, and the term aromatherapy usually implies massage with a range of aromatic plant extracts known as essential oils [13]. There are many types of CAM treatments for dialysis patients, such as thermomechanical stimulation [14], eugenol nanoemulsion [15], and padded dressing with lidocaine HCL [16]. Aromatherapy, also known as essential oil therapy, is also a type of CAM therapy. It is a natural therapy used to balance, regulate, and promote bodily and mental health with natural essential oils extracted from plants [17]. Several studies have shown that aromatherapy can improve patients’ anxiety, depression, sleep, arteriovenous fistula puncture pain, and symptoms of restless legs syndrome (RLS), promote physical and mental comfort, and improve patients’ quality of life [18–22].

Currently, aromatherapy is being utilized in maintenance hemodialysis (MHD) patients, but the outcomes are still controversial. In this study, a meta-analysis was used to systematically evaluate the intervention effects of aromatherapy on fatigue, sleep quality, arteriovenous fistula puncture pain, anxiety, and stress in hemodialysis patients to provide evidence-based recommendations for clinical practice.

## Methods

### Study protocol

This meta-analysis was conducted in accordance with the Preferred Reporting Items for Systematic Reviews and Meta-Analyses (PRISMA) guidelines [23] and registered in INPLASY (DOI: 10.37766/inplasy2022.8.0030).

### PICO question

We considered information regarding the effect of aromatherapy (I) on quality of life in MHD patients (P) with or without a comparator (C). Our goal was to assess the effect of any treatment outcome on patients’ quality of life (O), including sleep quality, fatigue, anxiety, symptoms of RLS, and arteriovenous fistula puncture pain.

### Search strategy

Two researchers (ZY and ZC) performed a comprehensive literature search, and 22 relevant studies that met all eligibility criteria were obtained. To examine all relevant randomized control trials (RCTs) regardless of publication status, we searched the PubMed, EMBASE, Scopus, Web of Science, and CNKI databases for articles published prior to 6 May 2022 (Table S1). The following keywords were used: ‘aromatherapy’, ‘aromatic essential oils’, ‘aromatic massage’, ‘inhaled aromatherapy’, ‘haemodialysis’, and ‘MHD’ Reference lists from the identified studies were also searched for potentially eligible articles. Preliminary publications were imported into EndNote X9.1 (Clarivate Analytics, Philadelphia, PA), duplicate records and irrelevant studies were removed, and appropriate studies with detailed classification were compiled.

## Selection criteria

**Figure 1.**
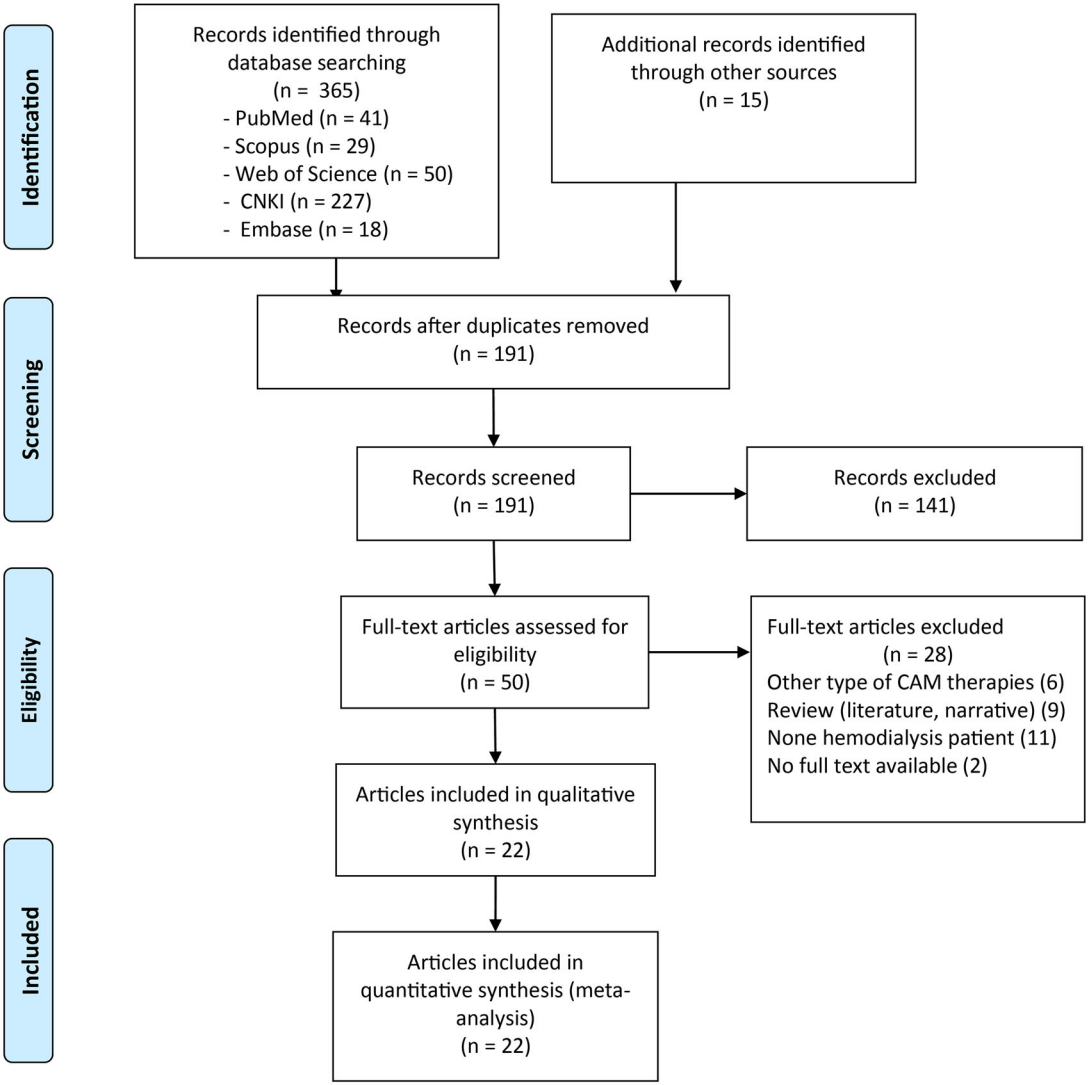
PRISMA 2020 flow diagram.

Two authors (ZY and ZC) independently carried out the primary review to search for trials that met the inclusion criteria. Any disagreements were solved by discussion or consultation with a third author (GWJ) (Figure 1 and Table S2).

The following criteria were used:

1. Only RCTs were included in the meta-analysis.
2. Studies that included participants who were on hemodialysis for at least 3 months, excluding patients with a history of kidney transplantation.
3. Studies in which the experimental group received aromatherapy, including inhalation of essential oils (olive, lavender, orange, almond, and rose) or massage with essential oils, and the control group received usual care or was given steaming in the same way.
4. Studies in which one of the following outcomes was included:

Fatigue: The Fatigue Severity Scale (FSS), the Brief Fatigue Inventory (BFI), and the Rhoten Fatigue Scale (RFS) were used for evaluation.

Sleep Quality: The Pittsburgh Sleep Quality Index (PSQI) and the Visual Analogue Scale (VAS) were used.

Arteriovenous fistula pain: The VAS and the Numeric Rating Scale (NRS) were used.

Anxiety: The Beck Anxiety Inventory (BAI), the State‐Trait Anxiety Inventory (STAI), the Hamilton Anxiety Rating Scale (HAM-A), the Depression Anxiety Stress Scale, the Depression Anxiety Stress Scale‐21 (DASS‐21), and the Hospital Anxiety and Depression Scale (HADS) were used.

Sleep/RLS: The PSQI and the RLS rating scale were used.

The main characteristics of the included studies are listed in Table 1.

**Table 1.** Main characteristics of the included studies.

Study	Country	Sample		Intervention		Outcome	Frequency and follow-up	Measure
		T	C	Treatment groups	Control groups			
Ahmady et al. [24]	Iran	30	30	Five drops of lavender oil	Five drops of distilled water	Fatigue	Three times a week for 30 min for 2 weeks	FSS
Ahmady et al. [24]^^	Iran	30	30	Five drops of orange oil	Five drops of distilled water	Fatigue	Three times a week for 30 min for 2 weeks	FSS
Kılıç Akça et al. [22]^^	Turkey	25	25	Lavender and tea tree oils	Standard care	Pain	Three times a week for 15 min for 8 weeks	VAS
Alireza et al. [25]	Iran	30	30	Three drops of lavender oil	Standard care	Sleep	During dialysis for 4 weeks	PSQI
Efe Arslan and Kılıç Akça [26]	Turkey	22	22	Lavender oil	Standard care	Distress, sleep	Three times a week for 10 min for 4 weeks	DT, PSQI
Arzu [27]	Turkey	17	17	Two drops of lavender oil	Standard care	Anxiety, sleep	30 min before sleep for 1 week	VAS, HAS
Bagheri-Nesami et al. [28]	Iran	30	30	Three drops of lavender oil	Standard care	Fatigue	three times a week for 10 min for 4 weeks	FSS
Barati et al. [20]	Iran	23	23	Rose water	Standard care	Anxiety	Three times a week for 10 min for 4 weeks	SAS
Chen et al. [29]	China	40	38	Chinese herbology incense therapy	Standard care	Anxiety, distress	Once a day for 30–60 min for 4 weeks	SAS, SDS
Dehkordi et al. [30]	Iran	30	30	Three drops of damask rose oil	Standard care	Depression, anxiety, and stress	During dialysis for one hour for 4 weeks.	DASS21
Mohammadpourhodki et al. [21]	Iran	35	35	10–15 cc of 1.5% lavender oil	Standard care	Pain	During dialysis for 20 min twice a day for 4 weeks	SF-36
Mohammadpourhodki et al. [21]	Iran	35	35	10–15 cc of 1.5% citrus Aurantium oil	Standard care	Pain	During dialysis for 20 min three times per week for 4 weeks	SF-36
Ghasemi et al. [19]	Iran	35	35	Aromatherapy massage	Standard care	RLS	Three times a week for 15 min for 3 weeks	RLS rating scale
Ghods et al. [31]	Iran	17	17	Three puffs of lavender oil	Three puffs of water	Pain	three times for 60 min, with an interval of at least 3 d	NRS
Mohammadali Hassanzadeh et al. [32]	Iran	35	35	Two drops of 5% lavender essential oil	Standard care	Fatigue	During dialysis and at home twice a day for 4 weeks	BFI
Huang [33]	China	42	40	1% lavender essential oil	Distilled water	Pain, anxiety	During dialysis for 1 h for 4 weeks	SAI, VAS
Imani et al. [34]	Iran	30	30	Ten drops of lavender oil	Standard care	Pain	During dialysis for 5 min for 4 weeks.	VAS
Imani et al. [34]	Iran	30	30	Ten drops of olive oil	Standard care	Pain	During dialysis for 5 min for 4 weeks	VAS
Karadag et al. [35]	Turkey	30	30	Two drops of lavender oil	Standard care	Anxiety, fatigue	During dialysis for 20 min for 4 weeks.	BAI, FSS
Kiani et al. [36]	Iran	35	35	Two drops of 5% lavender oil and sweet almond oil	Standard care	Anxiety	During dialysis for 20 min twice a day for 4 weeks	STAI
Muz and Taşcı [37]	Turkey	27	35	Sweet orange and lavender oil	Standard care	Fatigue, sleep	During dialysis for 2 min for 4 weeks	VAS, PSQI
Najafi et al. [38]	Turkey	30	30	Lavender essential oil	Standard care	Sleep	Not clear	PSQI
Oshvandi et al. [39]	Iran	35	35	10–15 cc of lavender oil	Standard care	Sleep	Three times a week for 30 min for 3 weeks	RLS rating scale
Oshvandi et al. [39]	Iran	35	35	10–15 cc of sweet orange oil	Standard care	RLS	Three times a week for 30 min for 3 weeks	RLS rating scale
Taşan et al. [40]	Turkey	30	30	Three drops of lavender and sweet almond oil	Standard care	Pain	During dialysis for 3–5 min for 4 weeks	VAS
Varaei et al. [41]	Iran	32	32	Inhalation aromatherapy	Standard care	Fatigue	Three times a week for 20 min for 16 weeks	RFS
Varaei et al. [41]	Iran	32	32	Aromatherapy massage	Standard care	Fatigue	Three times a week for 20 min for 16 weeks	RFS

BAI: Beck Anxiety Inventory; BFI: Brief Fatigue Inventory; DASS-21: Depression Anxiety Stress Scale-21; DT: Distress Thermometers; FSS: Fatigue Severity Scale; HAS: Hamilton Anxiety Scale; NRS: Numeric Rating Scale; PSQI: Pittsburgh Sleep Quality Index; RFS: Rhoten Fatigue Scale; RLS: Restless Legs Syndrome; SAI: State Anxiety Inventory; SAS: State Anxiety Scores; SDS: Self-rating Depression Scale; SF-36: Short Form Health Survey-36; STAI: State Trait Anxiety Inventory; VAS: Visual Analogue Scale

### Risk of bias assessment

The quality of each trial was evaluated independently by two authors (GWJ and ZC) according to the Cochrane quality criteria. Any disagreement between the authors (GWJ and ZC) was settled by discussion with a third author (ZY). Ethical approval was not needed, as this protocol did not involve any direct contact with patients. A risk of bias assessment of each methodological component was performed by each reviewer (Figure S1), and a weighted kappa value was calculated to examine agreement for each component (Table S3). An overall risk of bias assessment was also performed by each reviewer (Table S4). A weighted kappa value was calculated to examine agreement between reviewers for the overall study risk of bias assessment (Table S4).

### GRADE quality assessment

The overall quality of evidence was evaluated by two authors (Cong Zhang and Wen-Jun Gou) according to The Grading of Recommendations Assessment, Development and Evaluation (GRADE) criteria, evaluating the evidence on [42,43] (i) study limitations, (ii) inconsistency, (iii) imprecision, (iv) indirectness, and (v) publication bias. Any disagreement between the two authors was first resolved by discussion and then by consulting with a third author (Hang Mu) or the senior author (Yong Zhang). The results and the overall quality of evidence are presented in Table 2.

**Table 2.** Summary of findings.

Quality assessment							Summary of findings			
Outcomes	Study design	Risk of bias	Inconsistency	Indirectness	Imprecision	Publication bias	No. of patients		Absolute (95% CI)	Overall quality of evidence
							Treatment	Control		
Fatigue	RCT	Not serious	Serious	Not serious	Not serious	Not serious	184	192	−1.32 (−2.58 to −0.05)	⊕⊕⊕◯ Moderate
Pain	RCT	Not serious	Serious	Not serious	Not serious	Not serious	174	172	−1.98 (−3.02 to −0.95)	⊕⊕⊕◯ Moderate
Sleep	RCT	Not serious	Serious	Not serious	Not serious	Not serious	169	161	−1.52 (−2.38 to −0.67)	⊕⊕⊕◯ Moderate
Symptoms of RLS	RCT	Not serious	Serious	Not serious	Not serious	Not serious	70	70	−1.71 (−2.09 to −1.33)	⊕⊕⊕◯ Moderate
Anxiety	RCT	Not serious	Serious	Not serious	Not serious	Not serious	208	281	−1.58 (−2.07 to −1.08)	⊕⊕⊕◯ Moderate

GRADE Working Group grades of evidence.

High quality: Further research is very unlikely to change our confidence in the estimate of the effect.

Moderate quality: Further research is likely to have an important impact on our confidence in the estimate of the effect and may change the estimate.

Low quality: Further research is very likely to have an important impact on our confidence in the estimate of the effect and is likely to change the estimate.

Very low quality: We are very uncertain about the estimate.

### Data extraction

Two reviewers (Zhang Chong and Zhang Yong) independently extracted data from the same set of publications. The following information was extracted: author, year, country, sample size, intervention, outcome, intervention group, control group, frequency, follow-up, and method of measurement.

### Statistical analysis

STATA version 16.0 (Stata Corp LP, College Station, TX) was used to perform the statistical analyses. Odds ratios (ORs) with 95% confidence intervals (CIs) were used as the effect size measures of dichotomous data. Mean differences (MDs) with 95% CIs were computed for continuous data. A weighted fixed-effects model was used if there was no significant heterogeneity; otherwise, a random-effects model was used. Statistical heterogeneity was analyzed by I^2^ and χ^2^ statistics. The critical value for homogeneity was a *p* value less than 0.05. If there was significant heterogeneity, a sensitivity analysis was conducted to evaluate the consistency and quality of the results. Labbe plots and meta-regression were used for intuitive judgment of heterogeneity.

Heterogeneity was categorized as follows: low, *Ι*^2^ = 0–25%; medium, *Ι*^2^ = 25–50%; high, *Ι*^2^ = 50–75%; and strong, *Ι*^2^ = 75–100%. An *Ι*^2^ of less than 50% was considered to represent acceptable heterogeneity. *p* Values were determined using the *χ*^2^ test, and the results were considered statistically significant at *p* < 0.05 for all included studies.

## Results

### Study selection

A total of 380 studies were identified during the initial search after excluding duplicate records (*n* = 35). A total of 191 articles were retained after title/abstract screening (excluding 189 records). An additional 141 articles were excluded because they had unrelated titles. Following a detailed evaluation, 28 articles were excluded because three were review articles, six used other types of CAM therapies, nine were reviews, eleven included nonhemodialysis patients, and two had no full text available. Ultimately, 22 studies [19,21,22, 26–41,44] fulfilled the inclusion criteria for this meta-analysis. The literature screening process and results are shown in Figure 1.

### Fatigue

**Figure 2.**
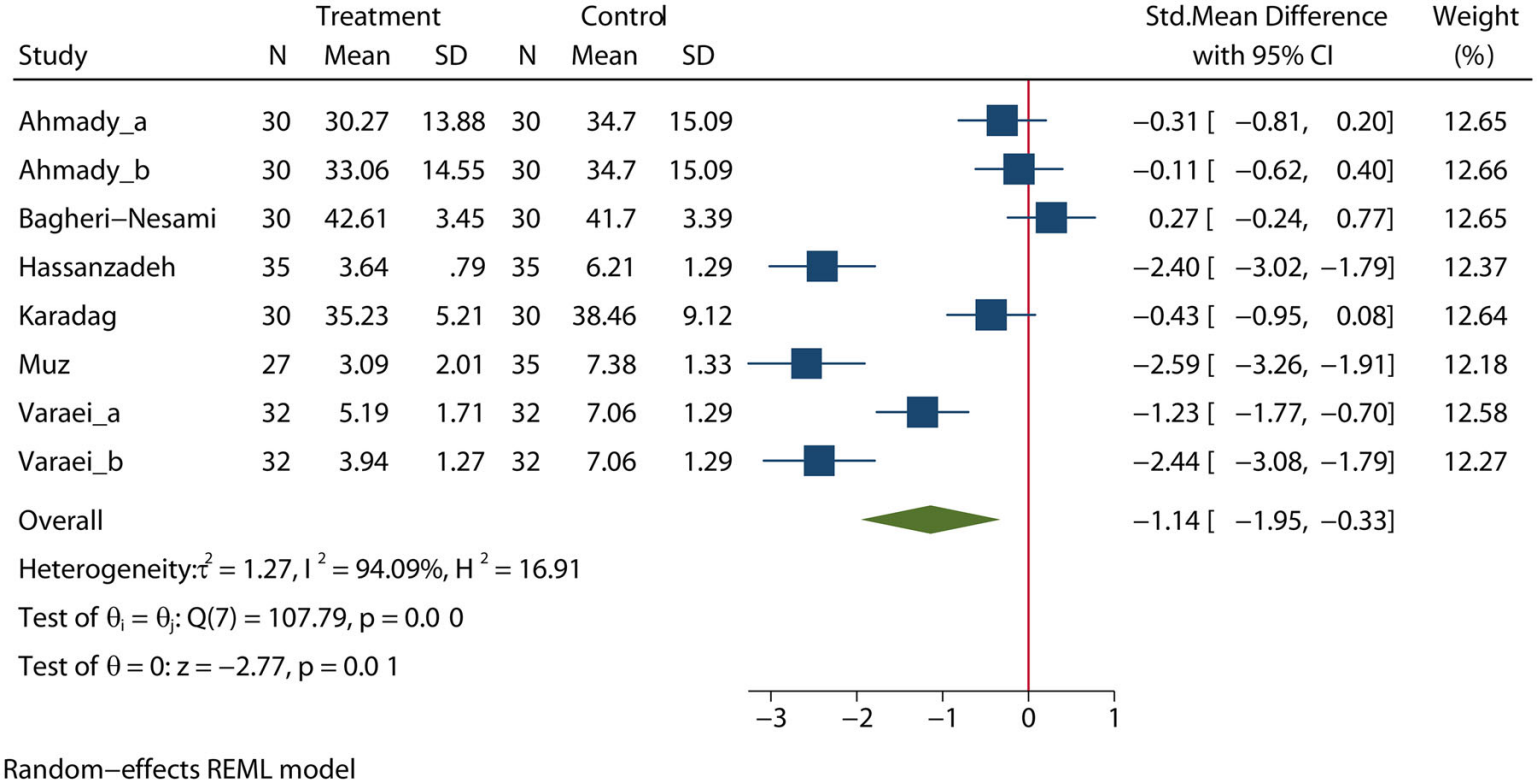
Effect of aromatherapy on fatigue in MHD patients.

Six articles [24,28,32,35,37,41] reported on the effects of aromatherapy on fatigue in MHD patients. There was high heterogeneity among these studies (*I*^2^ = 94%); therefore, a random-effects model was used for the analysis. The analysis indicated a statistically significant difference in fatigue between the experimental and control groups (SMD = −1.14, 95% CI [−1.95, −0.33], *p* = 0.01], as shown in Figure 2.

A subgroup analysis also suggested that aromatherapy treatment significantly reduced fatigue in Iranian studies, while there was no significant treatment effect on fatigue in Turkish studies (Figure S2). The meta-regression *via* bubble plot assessment revealed no significant heterogeneity in the publication year (*p* = 0.749, Figure S3) or publication country (*p* = 0.646, Figure S4); however, study size was a potential major source of heterogeneity (*p* = 0.008, Figure S5). A sensitivity analysis was performed to evaluate the stability of our results (Figure S6). The results suggested that no individual study significantly impacted the pooled OR, indicating that the results were statistically robust.

### Quality of sleep

**Figure 3.**
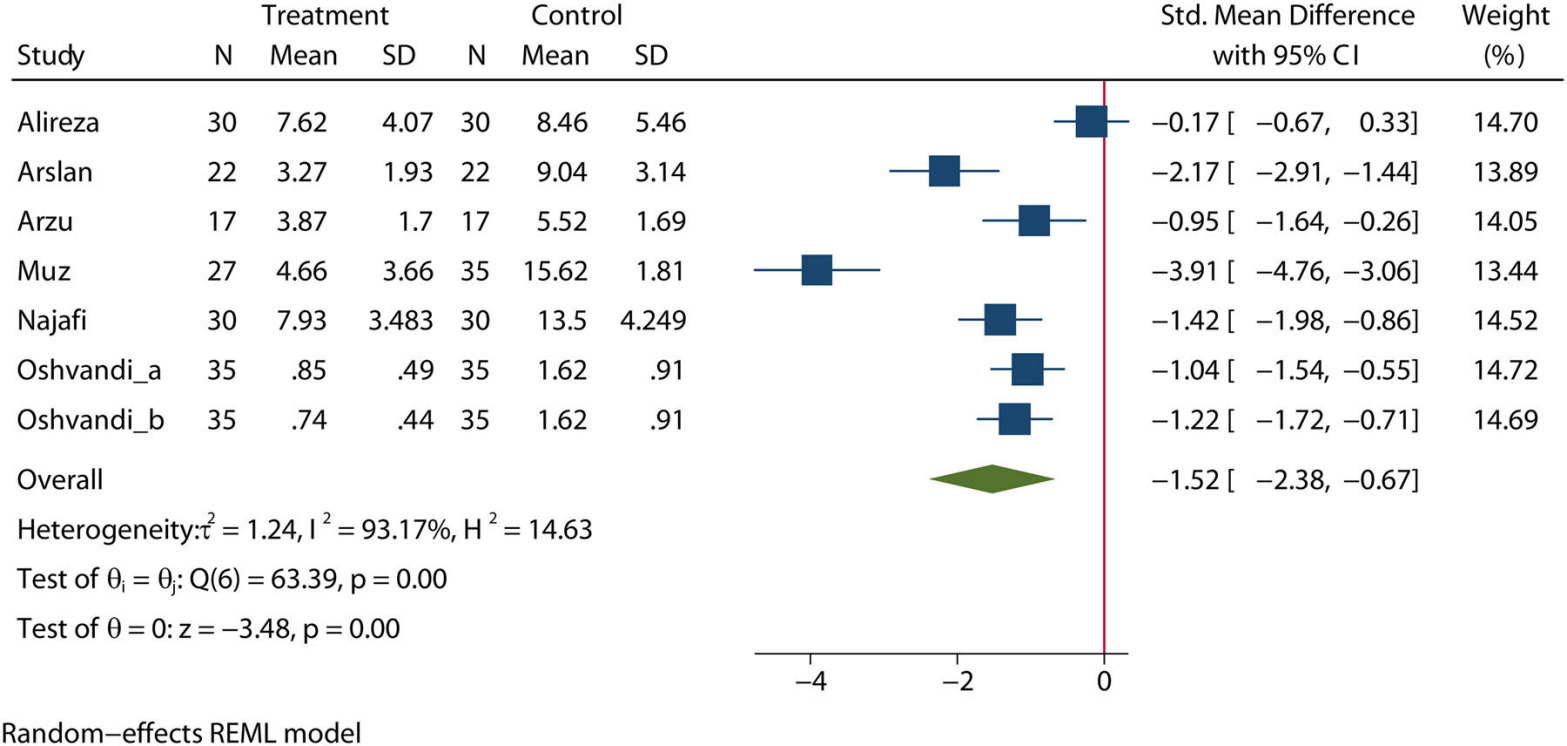
Effect of aromatherapy on sleep quality in MHD patients.

Six articles [25–27,38,37,39] reported on the effects of aromatherapy on sleep quality in MHD patients. The heterogeneity among these studies was high (*I*^2^ = 93.1%); therefore, a random-effects model was used for the analysis. The analysis indicated a statistically significant difference in sleep quality between the experimental and control groups (SMD= −1.52, 95% CI [−2.38, −0.67], *p* < 0.01], as shown in Figure 3. These findings were confirmed in a subgroup analysis (Figure S7).

As there was substantial heterogeneity, a sensitivity analysis was performed to assess the influence of each individual study (Figure S8). No significant publication bias was found for the publication year (*p* = 0.979, Figure S9), study size (*p* = 0.676, Figure S10), or publication country (*p* = 0.103, Figure S11). A sensitivity analysis was performed to evaluate the stability of our results (Figure S12). The results suggested that no individual study significantly influenced the pooled OR, indicating that the results were statistically robust.

### Arteriovenous fistula puncture pain

**Figure 4.**
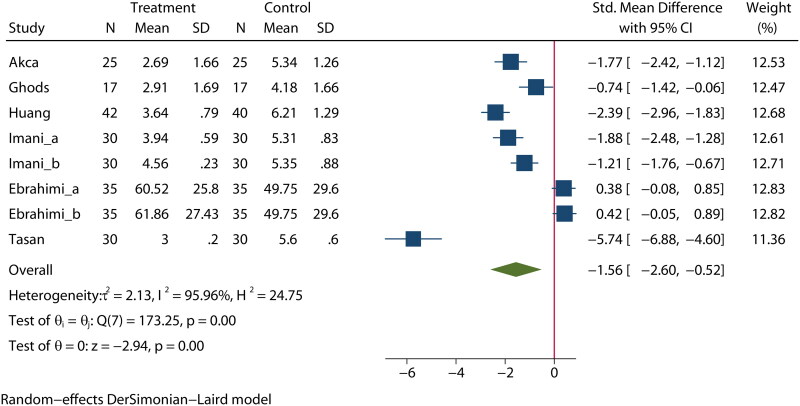
Effect of aromatherapy on pain in MHD patients.

Six articles [21,22,31,33,34,40] reported on the effects of aromatherapy on arteriovenous fistula puncture pain in MHD patients. The heterogeneity among these studies was high (*I*^2^ = 95.4%); therefore, a random-effects model was used for the analysis. The results revealed that there was a statistically significant difference in arteriovenous fistula puncture pain between the experimental group and the control group (SMD = −1.56, 95% CI [−2.60, −0.52], *p* < 0.01], as shown in Figure 4.

A subgroup analysis found that there was a significant reduction in arteriovenous fistula puncture pain by treatment in studies conducted in China and Turkey, while there was no significant reduction in Iranian studies (Figure S13). A meta-regression by bubble plot assessment revealed no significant heterogeneity in the publication year (*p* = 0.638, Figure S14), study size (*p* = 0.901, Figure S15), or publication country (*p* = 0.305, Figure S16). A sensitivity analysis was performed to evaluate the stability of our results (Figure S17). The results suggested that no individual study significantly affected the pooled OR, indicating that the results were statistically robust.

### Anxiety

**Figure 5.**
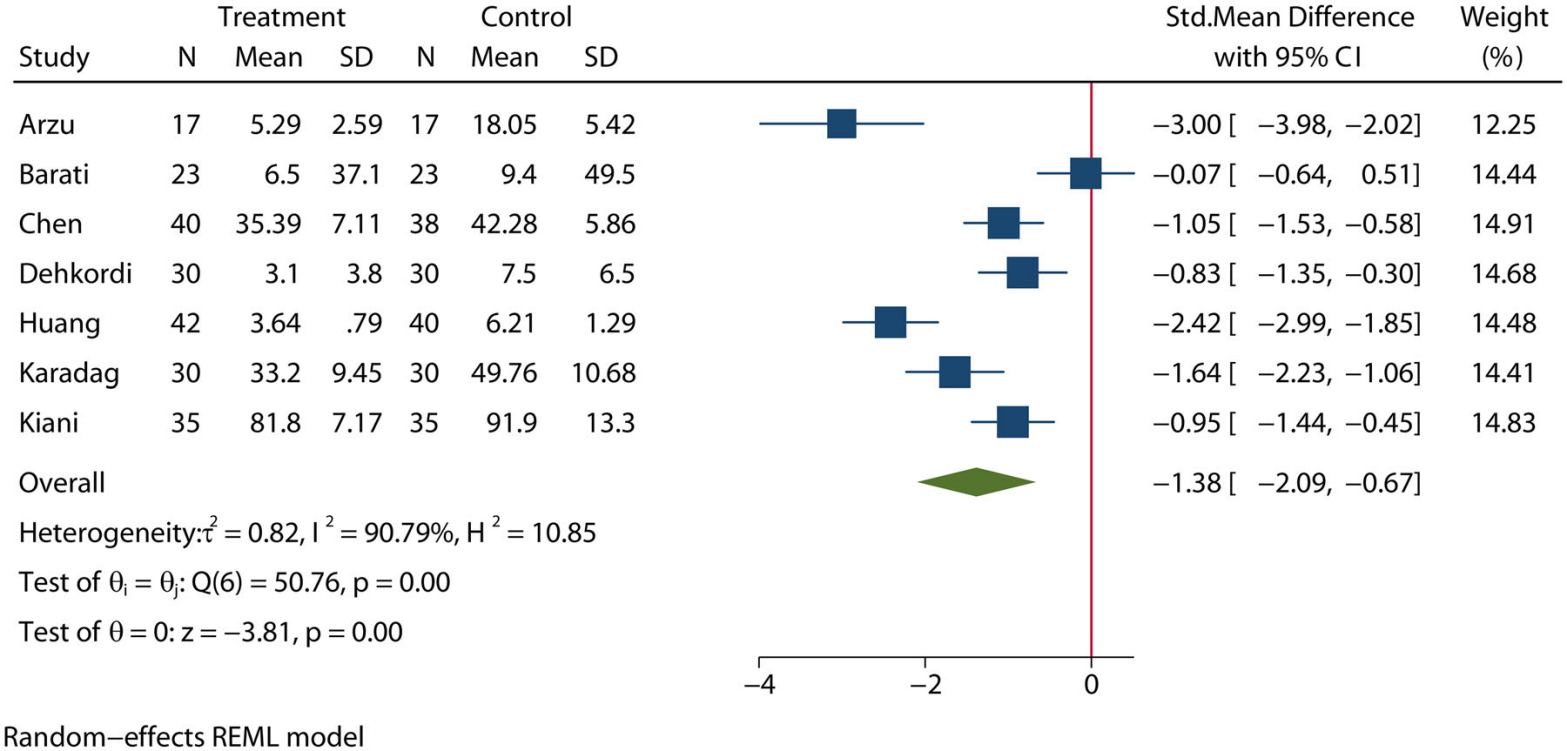
Effect of aromatherapy on anxiety in MHD patients.

Seven articles [20,27,29,30,33,35,36] reported on the effects of aromatherapy on anxiety in MHD patients. The heterogeneity among studies was high (*I*^2^ = 90.8%); therefore, a random-effects model was used for the analysis. The results indicated that there was a significant difference in anxiety between the experimental and control groups (SMD = −1.38, 95% CI [−2.09, −0.67], *p* < 0.01], as shown in Figure 5.

A subgroup analysis also suggested that there were significant reductions in measures of anxiety in studies conducted in China and Turkey, while there were no significant differences in these measures in Iranian studies (Figure S18). The meta-regression by bubble plot assessment revealed no significant heterogeneity for the publication year (*p* = 0.638, Figure S19), study size (*p* = 0.901, Figure S20), or publication country (*p* = 0.647, Figure S21). A sensitivity analysis was performed to evaluate the stability of our results (Figure S22). The results suggested that no individual study significantly impacted the pooled OR, indicating that the results were statistically robust.

### Restless legs syndrome

**Figure 6.**
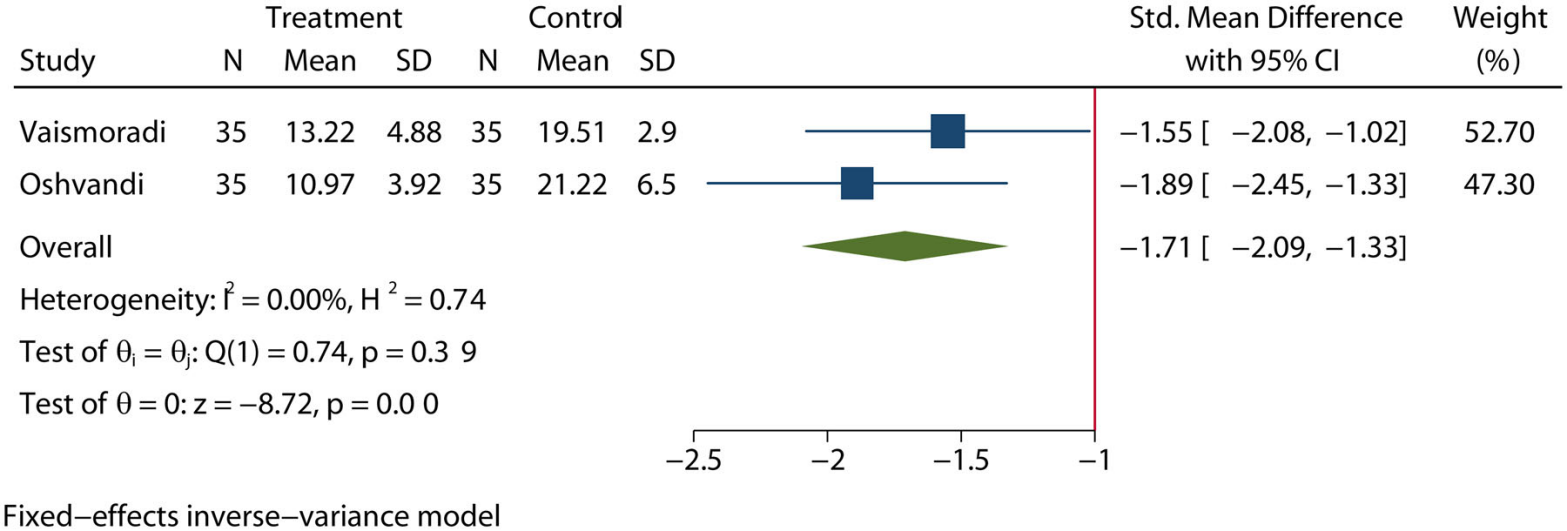
Effect of aromatherapy on symptoms of RLS in MHD patients.

Only two studies [19,39] reported the presence of RLS in MHD patients. A fixed-effects model was used, as the heterogeneity was low (*I*^2^ = 0%). Aromatherapy treatment led to a statistically significant decrease in the symptoms of RLS in the experimental group (SMD = −1.71, 95% CI [−2.09, −1.33], *p* < 0.01) compared to the control group (Figure 6).

## Discussion

The Western perspective on health care focuses on medications for the treatment of health care conditions. It is common for pain to be treated with various levels of opioids and for patients to receive prescriptions for medications at each physician visit. Sadly, over time, the use of opioids and anti-anxiety medications often leads to drug addiction. Therefore, a search for alternative medicine has begun. The Eastern perspective on health care focuses on alternative methods. Westerners have found many alternative methods to treat medical conditions, such as yoga, Pilates, mindfulness meditation, acupuncture, and scented oil massage. The pendulum began to swing from the Western medication approach to the Eastern holistic approach. Aromatherapy emerged and has been embraced as an alternative medicine for many medical conditions [12].

This meta-analysis was conducted to determine the effects of aromatherapy on hemodialysis complications from the inception of the databases to 6 May 2022. The method of treatment used most often was inhalation aromatherapy, and the most common aroma used was lavender.

The results of this study indicated that aromatherapy may reduce fatigue in patients on MHD. Among the various physical and psychological symptoms experienced by hemodialysis patients, the incidence of fatigue is the highest, accounting for 65.6∼91.0% [26]. Fatigue not only seriously impacts the quality of life of MHD patients but also increases the risk of cardiovascular events, which can influence a patient’s prognosis. Kang and Lee et al. [45,46] demonstrated that the type of essential oil may impact the effect of the intervention. A reason for this finding may be that compound essential oils have more components than single essential oils, leading to more pronounced physiological effects. Aromatic essential oils improve blood flow, relieve pain, relax muscles, and reduce fatigue by blocking brain impulses, endorphins, serotonin, and dopamine, thereby reducing sensitivity and muscle stiffness. The results of this study are inconsistent with those of Bagheri‐Nesami et al. [47], which may be related to many factors, including the intervention time, treatment dose and concentration, and treatment mode. Varaei et al. [41] found that the treatment effect of aromatic essential oil massage on the symptoms of MHD patients was greater than that of aromatic inhalation, although the findings of this study require further confirmation. Yangoz et al. also found that aromatherapy may help to reduce the severity of fatigue in adults receiving hemodialysis, but they did not analyze the broader effects of aromatherapy on dialysis patients [48].

It has been suggested that insomnia is associated with irregular physiological arousal. Patients with insomnia have been shown to have increased abnormal hormone secretion, whole body and brain metabolic activation, and sympathetic nervous system activation during sleep [49]. Investigations have shown that 30–80% of patients on MHD experience insomnia [44,50]. Goel et al. found that lavender was able to increase parasympathetic activity and the percentage of deep- or slow-wave sleep [51]. Lavender use also led to an increase in stage 2 (light) sleep and a decrease in both rapid eye movement (REM) sleep and the amount of time elapsed between first falling asleep and waking up (waking up after sleep onset) in females, with opposite effects found in males. Given these findings, it was concluded by the researchers that lavender may induce mild sedation and promote sleep [52]. These results indicate that aromatherapy may improve the sleep quality of MHD patients. However, high heterogeneity exists among studies for this index, which may be related to the essential oil concentration, treatment dose, and intervention duration, among other factors; thus, the results need to be confirmed by future studies.

Approximately, 48% of MHD patients experience fistula puncture-related pain and more than one-fifth of them find this pain to be intolerable [40]. Therefore, pain relief may increase both patients’ acceptance of the dialysis procedure and their quality of life. Essential oils used in aromatherapy are often applied with pressure, produce a cold sensation on the skin, and possess a pleasant scent that can relieve acute pain [53–55]. Lavender oil (*Lavandula officinalis*) provides acute pain control and produces sedative and local anesthetic effects, whereas tea tree oil (*Melaleuca alternifolia*) can improve skin conditions [56,57]. This study evaluated the effectiveness of aromatherapy in reducing the acute pain associated with catheter or arteriovenous fistula insertion in HD patients. Therefore, it is recommended that aromatherapy be used for MHD patients to manage pain development during vascular access.

Anxiety is a complex phenomenon associated with behavioral, psychological, physical, and psychiatric symptoms in MHD patients [36]. Hmwe et al. [58] found that the incidence of anxiety in MHD patients was 12–52%. Previous studies reported that aromatherapy, integrated with other treatment methods, was an effective method in the management of anxiety that also increased the quality of life of patients [59,60]. In this study, aromatherapy was observed to have an important role in managing HD patients’ frequent anxiety and in enhancing their quality of life. For these reasons, aromatherapy, as an integrative treatment method for relieving pain and managing anxiety, can be used in the treatment of MHD patients.

UP is one of the most bothersome symptoms among CKD patients. The pathophysiology of UP remains elusive, resulting in limited treatment options [61]. A recently proposed treatment for pruritus is the use of aromatherapy. Shahgholian et al. showed that aromatherapy can significantly relieve pruritus in MHD patients [62]. However, currently, there appears to be a paucity of data on its use. The generalization and application of this method still depend upon more accurate and more comprehensive studies in the field.

## Strengths and limitations

First, our meta-analysis was supervised by strict quality control evaluated by Cohen’s kappa coefficient (κ = 0.92), which showed a high degree of agreement. Second, in our study, 22 RCTs with more than 1300 participants were included, and a comprehensive and thorough assessment of the risks of publication bias, sensitivity analyses, subgroup analyses, Begg’s tests, Egger’s tests, and meta-regression analyses were conducted to ensure that the results were reliable. We attempted to be inclusive and transparent in this manuscript in terms of our methods, including all origins of software and websites. Third, we refined our analyses in strict accordance with the PICOS principle. Specifically, we conducted a comprehensive evaluation of the effect of aromatherapy on quality of life in MHD patients, including sleep quality, fatigue, anxiety, symptoms of RLS, and arteriovenous fistula puncture pain.

However, this study has some limitations. First, this study only included studies published in Chinese and English, which may result in publication bias. Second, there were a limited number of RCTs included in this study; thus, the findings need further substantiation. Third, there was significant heterogeneity among studies, which may have stemmed from the essential oil types, concentrations, administration routes, measurement tools, and intervention frequencies and durations that were utilized.

## Conclusion

Aromatherapy is a novel complementary and alternative therapy that may be used to improve fatigue, sleep quality, and anxiety in patients on MHD, although its use does not significantly reduce arteriovenous fistula puncture pain. High-quality multicenter trials with larger sample sizes are needed to further explore the intervention effect of aromatherapy on complications associated with MHD. Future research should explore the use of aromatic massage and aroma diffusion in MHD patients and aim to investigate the effects of different oil types, dosing protocols, concentrations, frequencies, and practices. Furthermore, future studies should examine whether the effects of aromatherapy vary by patient sex. Finally, to provide more reliable evidence for clinical practice, study follow-up times should be extended to explore the long-term impacts of aromatherapy use on symptoms in MHD patients.

## Supplementary Material

Supplemental Material

Supplemental Material

## Data Availability

Data are available on reasonable request from the authors.
