# Supplementary material for: Effect of aromatherapy on quality of life in maintenance hemodialysis patients: a systematic review and meta-analysis
Source: Ren Fail. 2023 Mar 12;45(1):2164202. doi: 10.1080/0886022X.2022.2164202 (PMC10013488; doi:10.1080/0886022X.2022.2164202)
Supplement: Supplemental Material [file IRNF_A_2164202_SM4936.pdf]

Table S1. Search strategy

Database	No	PICOS	Strategy
PubMed	#1	P	Renal Dialysis [MeSH] OR Dialysis OR Hemodialysis OR Haemodialysis OR ESRD OR Hemodialyses OR Peritoneal Dialysis
	#2	I	Aromatherapy [MeSH] OR Aromatic essential oil OR Aromatic massage OR Inhalational aroma OR lavender oil OR orange oil OR damask rose oil OR sweet almond oil
	#3	C	NA
	#4	O	NA
	#5	S	(randomized controlled trial [Publication Type] OR randomized [TIAB] OR randomized [TIAB] OR placebo[TIAB]) NOT (Review[Publication Type]) NOT (meta-analysis[Publication Type]) NOT (Comment[Publication Type]) NOT (Letter[Publication Type])
	#6 =	NA	((Renal Dialysis [MeSH] OR Dialysis OR Hemodialysis OR Haemodialysis OR ESRD OR Hemodialyses OR Peritoneal Dialysis) AND (Aromatherapy [MeSH] OR Aromatic essential oil OR Aromatic massage OR Inhalational aroma OR lavender oil OR Olive oil OR orange oil OR damask rose oil OR sweet almond oil)) AND ((randomized controlled trial[Publication Type] OR randomized[TIAB] OR randomised[TIAB] OR placebo[TIAB]) NOT (Review[Publication Type]) NOT (meta-analysis[Publication Type]) NOT (Comment[Publication Type]) NOT (Letter[Publication Type]))
Embase	#7	P	'Dialysis'/exp OR 'hemodialysis':ab,ti OR 'ESRD':ab,ti OR ‘Haemodialysis’:ab,ti OR ‘Renal Dialysis’:ab,ti OR 'Renal Dialysis':ab,ti OR ' Peritoneal Dialysis ':ab,ti
	#8	I	'Aromatherapy'/exp OR 'Aromatic essential oil':ab,ti OR 'Aromatic massage':ab,ti OR 'Inhalational aroma':ab,ti OR 'lavender oil':ab,ti OR 'Olive oil':ab,ti OR 'orange oil':ab,ti OR 'damask rose oil':ab,ti OR 'sweet almond oil':ab,ti
	#9	C	NA
	#10	O	NA
	#11	S	'randomized controlled trial'/exp NOT review:it
	#12	NA	('Dialysis'/exp OR 'hemodialysis':ab,ti OR 'ESRD':ab,ti OR ‘Haemodialysis’:ab,ti OR ‘Renal Dialysis’:ab,ti OR 'Renal Dialysis':ab,ti OR ' Peritoneal Dialysis ':ab,ti) AND ('Aromatherapy'/exp OR 'Aromatic essential oil':ab,ti OR 'Aromatic massage':ab,ti OR 'Inhalational aroma':ab,ti OR 'lavender oil':ab,ti OR 'orange oil':ab,ti OR 'damask rose oil':ab,ti OR 'sweet almond oil':ab,ti) AND ('randomized controlled trial'/exp NOT review:it)
Web of Science	#13	P	((((TS=(Renal Dialyses)) OR TS=(Hemodialysis)) OR TS=(Hemodialyses)) OR TS=(ESRD)) OR TS=(Dialysis)
	#14	I	((((((TS=(Aromatherapy)) OR TS=(Aromatic essential oil)) OR TS=(Aromatic massage)) OR TS=(lavender oil)) OR TS=(orange oil)) OR TS=(damask rose oil)) OR TS=(sweet almond oil)
	#15	C	NA
	#16	O	NA
	#17	S	ALL=(randomized controlled trial)
	#18	NA	#13 AND #14 AND #17

Abbreviation: NA: not applicable

Table S2. Inclusion/exclusion criteria of literature

PICOS	Inclusion	Exclusion
P	Adults participants (≥18 y) were on dialysis for at least 3 month, irrespective of age, gender, and race.	Children, pregnant women, and patients with a history of kidney transplantation were excluded
I	The experimental group received aromatherapy, including inhalation of essential oils (lavender, orange, almond, rose) or massage with essential oils	Not available.
C	The control group used usual care or was given steaming in the same way.	Not available.
O	One of the following outcomes must have been included: Fatigue, Sleep Quality, Arteriovenous fistula pain, Anxiety, Sleep Restless Leg Syndrome.	Not available.
S	RCT irrespective of blinding or arm	1) Articles without peer reviewed or unpublished<br>2) Studies that were repeatedly published or had qualitative outcomes<br>3) Quasi-experimental studies and observational studies

Table S3. Weighted Kappa Measurements to Assess Agreement Between Reviewers in Rating Quality of Methodology of Included Trials

	Random generation	Allocation concealment	Blinding	Incomplete outcome data	Selective reporting	Other bias
**κ**	1.00	0.93	0.60	0.84	0.92	0.68

Table 4. Overall Risk of Bias Assessment for Each Study

Study	Overall Quality Assessment
Ahmady 2019	High
Akça 2021	Unclear
Alireza 2016	High
Arslan 2020	Low
Arzu 2018	High
Bagheri-Nesami 2016	High
Barati 2016	Unclear
Chen 2019	Unclear
Dehkordi 2017	Unclear
Ebrahimi 2020	Low
Ghasemi 2021	Unclear
Ghods 2015	Unclear
Hassanzadeh 2018	Unclear
Huang 2015	Unclear
Imani 2021	Low
Karadag 2019	Unclear
Kiani 2016	Unclear
Muz 2017	Unclear
Najafi 2014	Unclear
Oshvandi 2021	Unclear
Taşan 2019	Unclear
Varaei 2020	High
κ	0.92

∗Overall risk of bias assessment was made for each study. Weighted kappa for agreement between reviewers is shown. A rating of “high” indicated the study had an increased risk of bias whereas a rating of “low” indicated the study had minimal risk of bias. Studies received a rating of “unclear” if reviewers were unable to determine risk of bias.
