# Supplementary material for: Effect of aromatherapy on quality of life in maintenance hemodialysis patients: a systematic review and meta-analysis
Source: Ren Fail. 2023 Mar 12;45(1):2164202. doi: 10.1080/0886022X.2022.2164202 (PMC10013488; doi:10.1080/0886022X.2022.2164202)

Fig S1 Assessment of risk of bias in included studies.

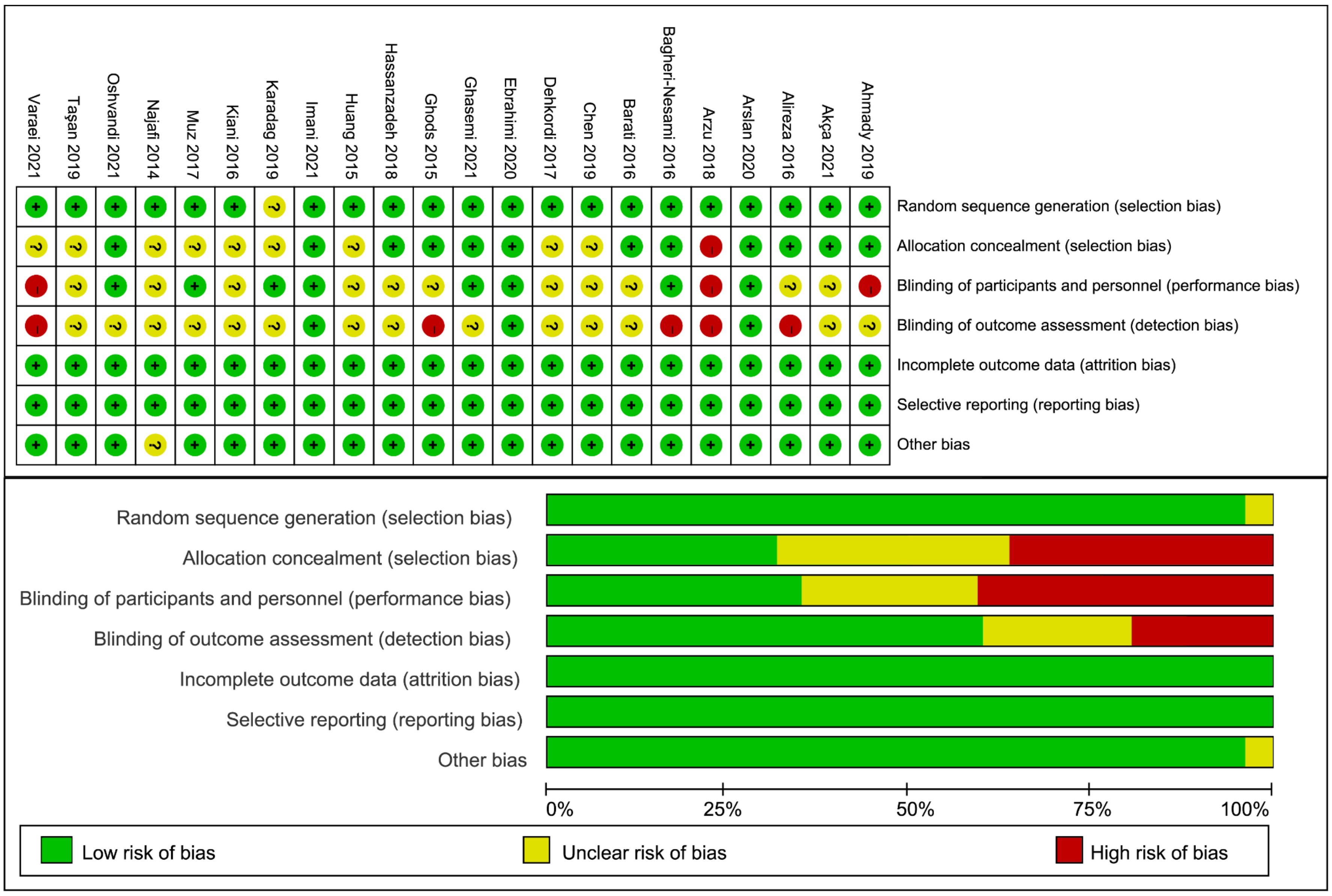

Fig S2 Subgroup analysis of aromatherapy on fatigue in MHD patients.

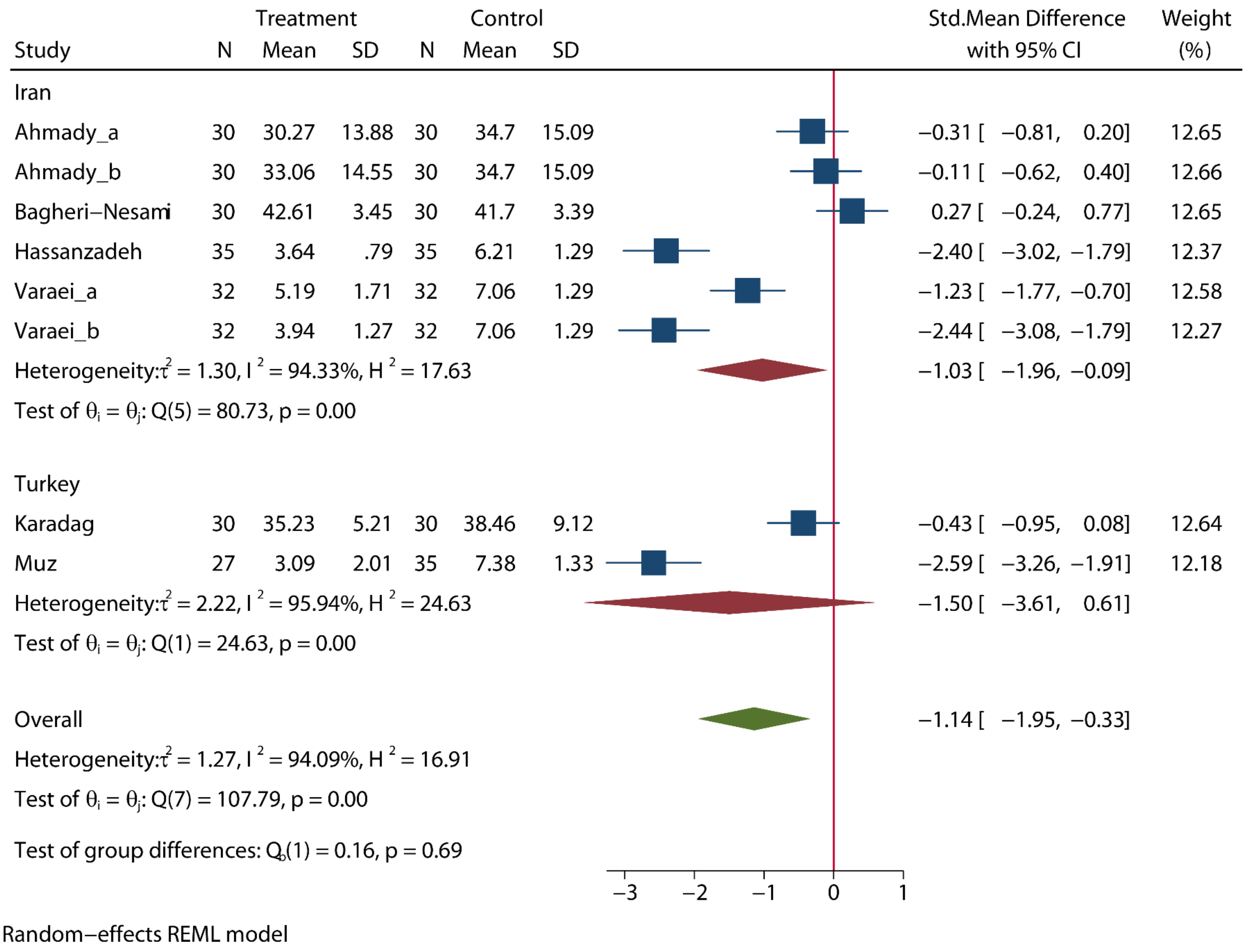

Information Classification: General

Fig S3 Meta-regression of publication year of fatigue in MHD patients.

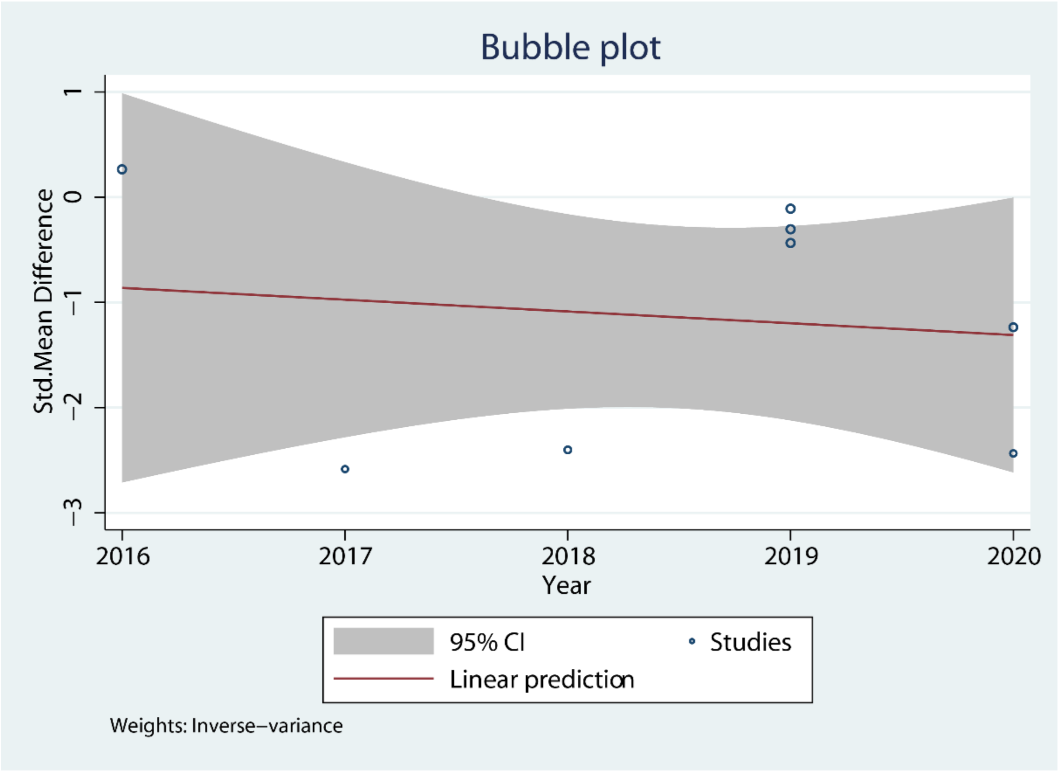

Fig S4 Meta-regression of publication countrys of fatigue in MHD patients.

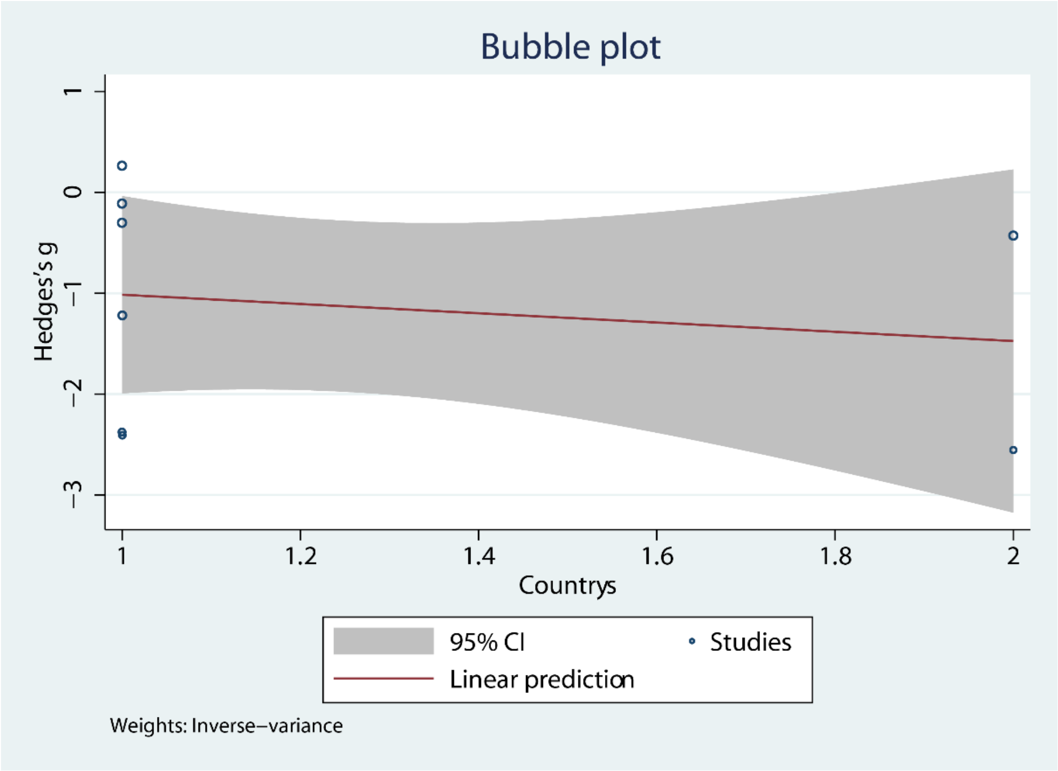

Fig S5 Meta-regression of study size of fatigue in MHD patients.

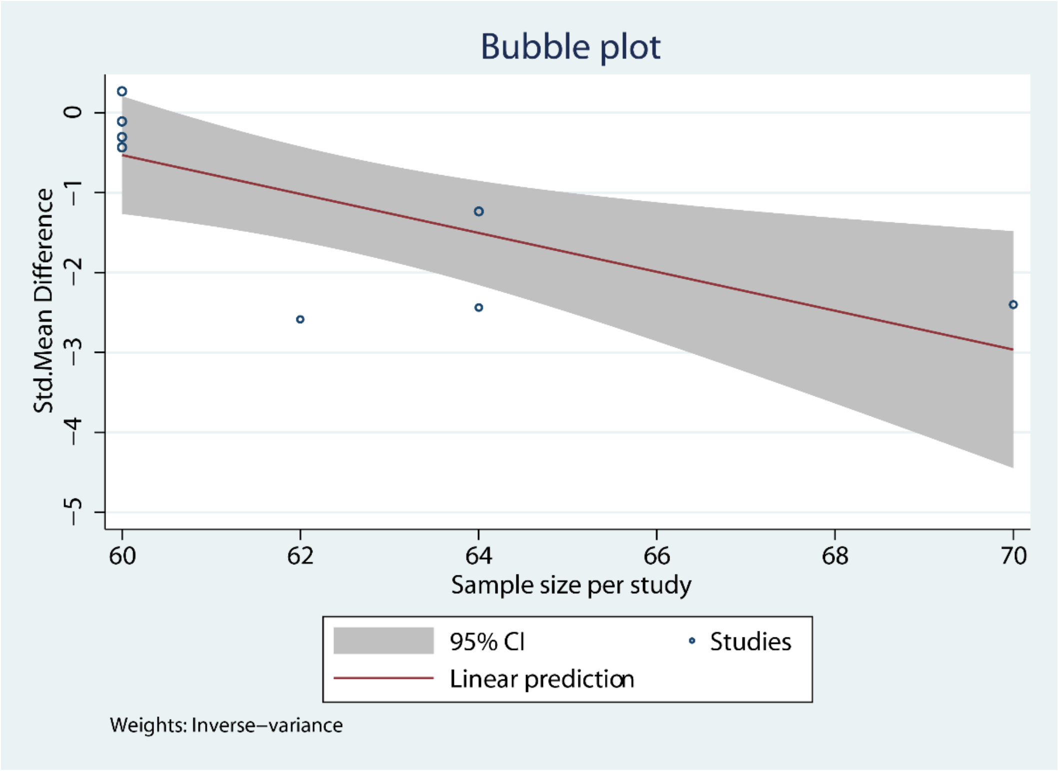

Fig S6 Sensitivity analysis for fatigue in MHD patients.

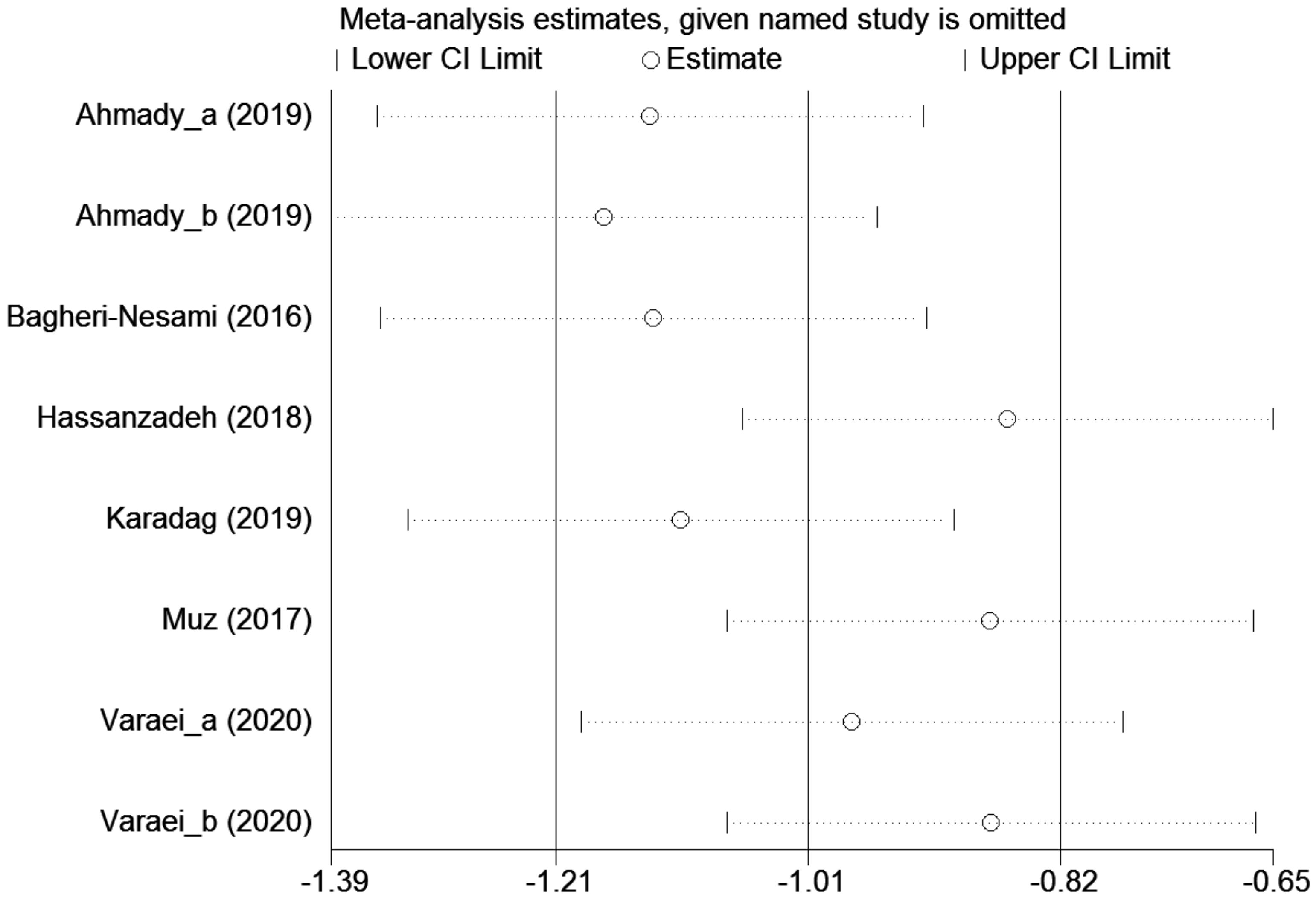

Fig S7 Subgroup analysis of sleep quality in MHD patients.

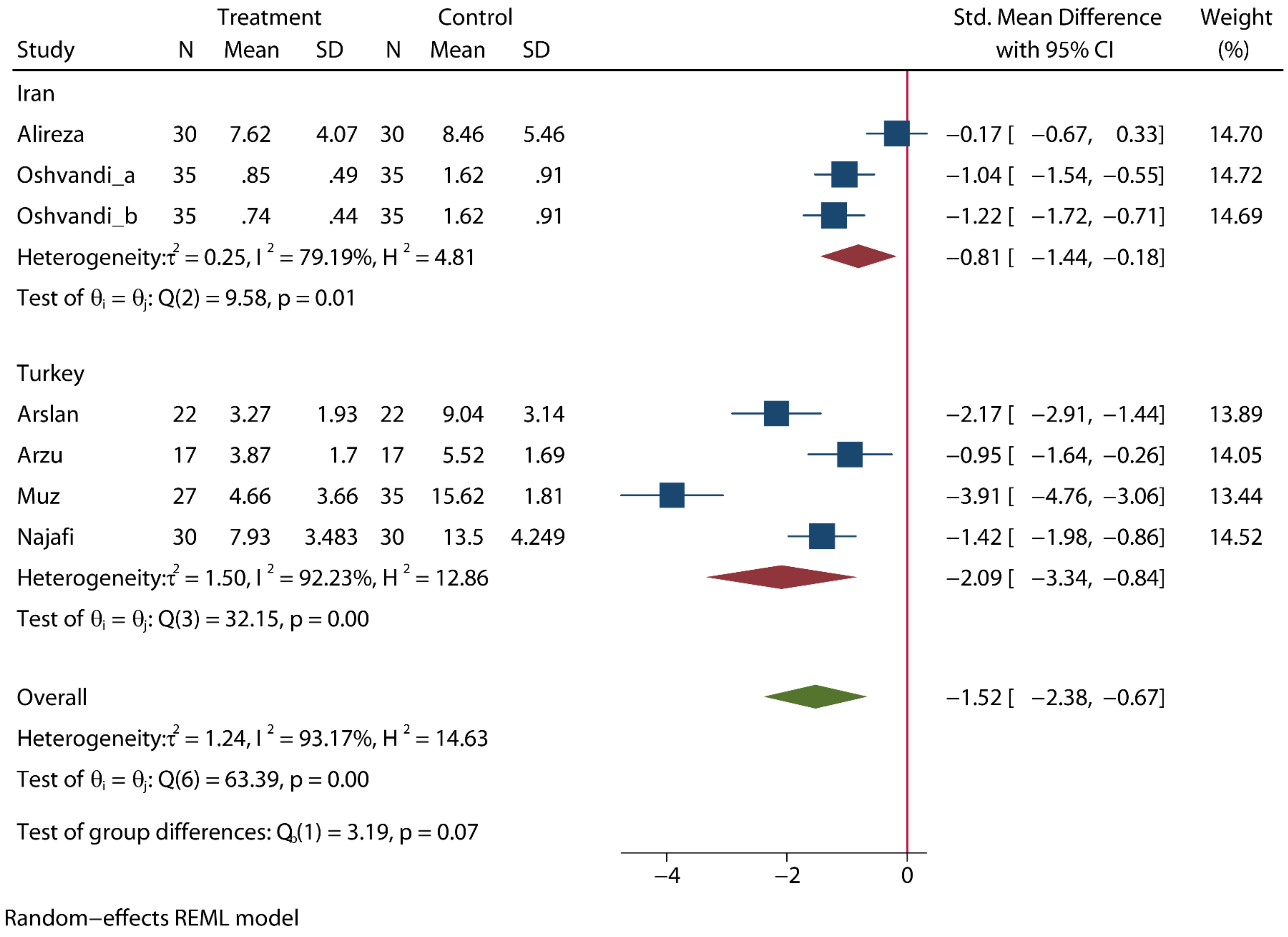

Information Classification: General

Fig S8 Sensitivity analysis of sleep quality in MHD patients.

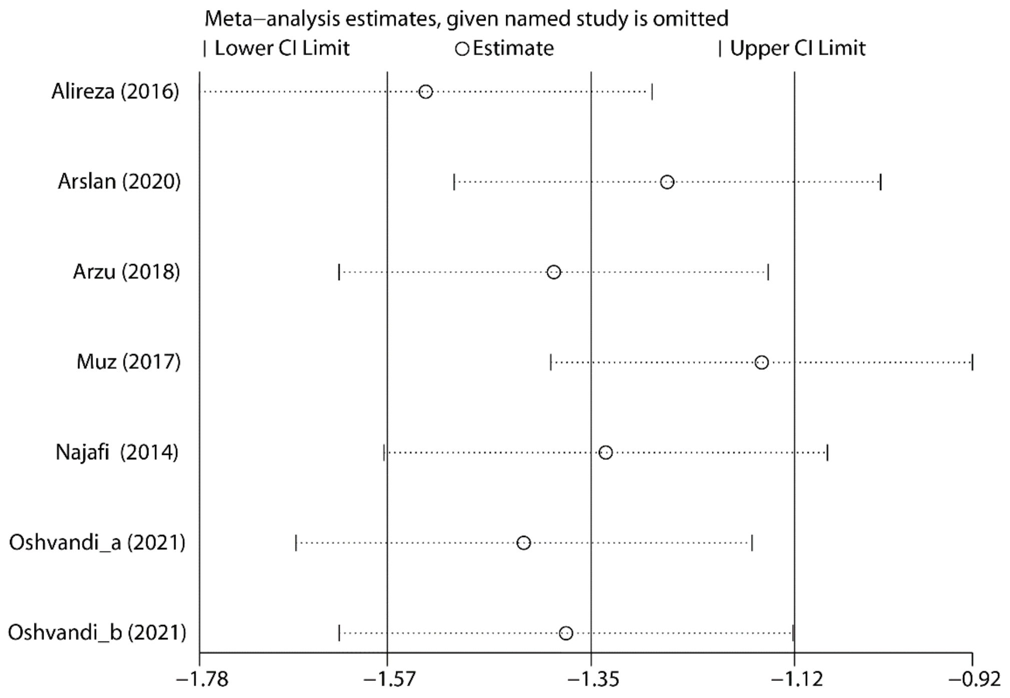

Fig S9 Meta-regression of publication year of sleep quality in MHD patients.

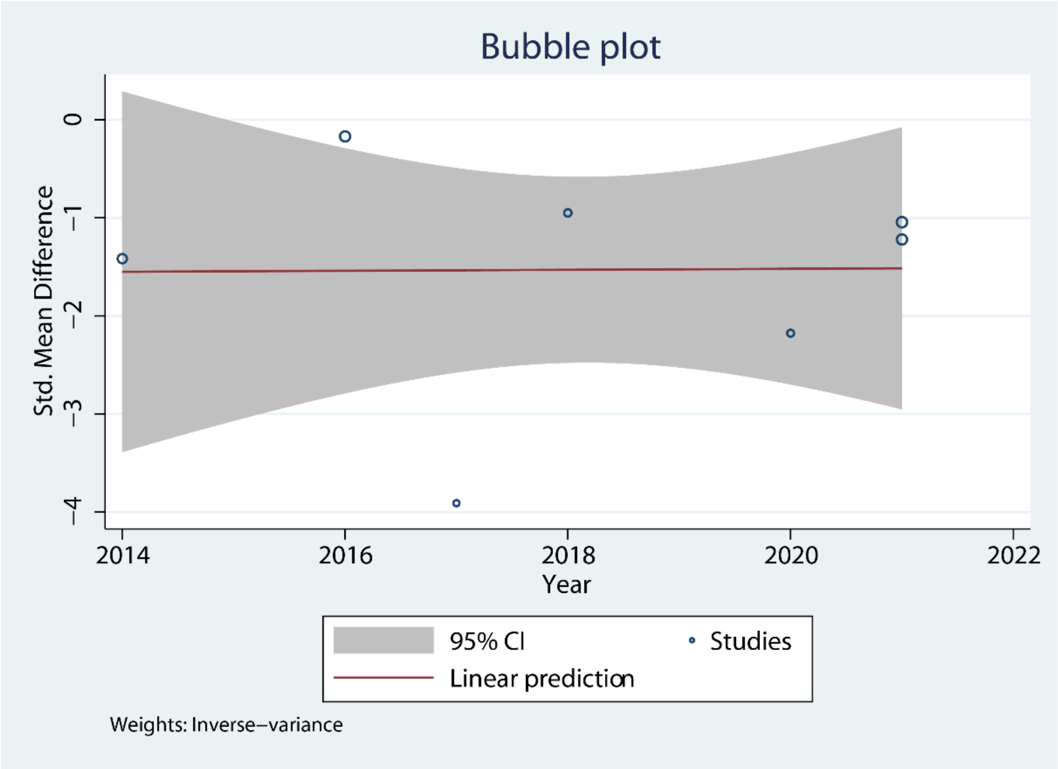

Fig S10 Meta-regression of study size of sleep quality in MHD patients.

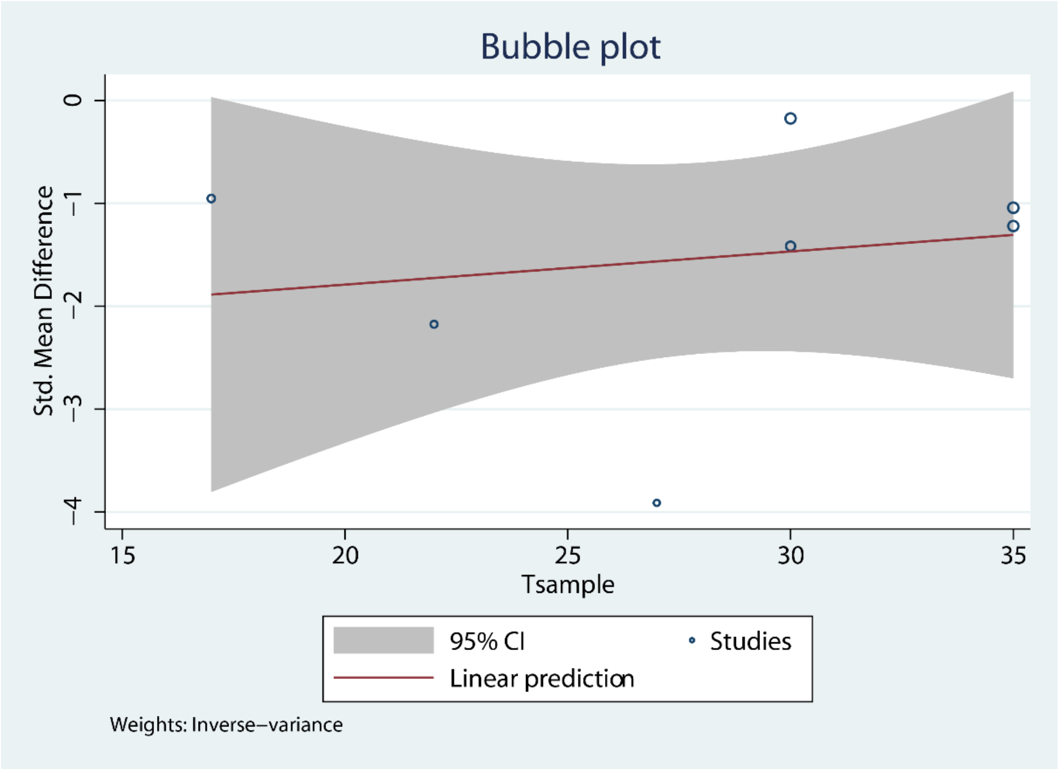

Information Classification: General

Fig S11 Meta-regression of publication country of sleep quality in MHD patients.

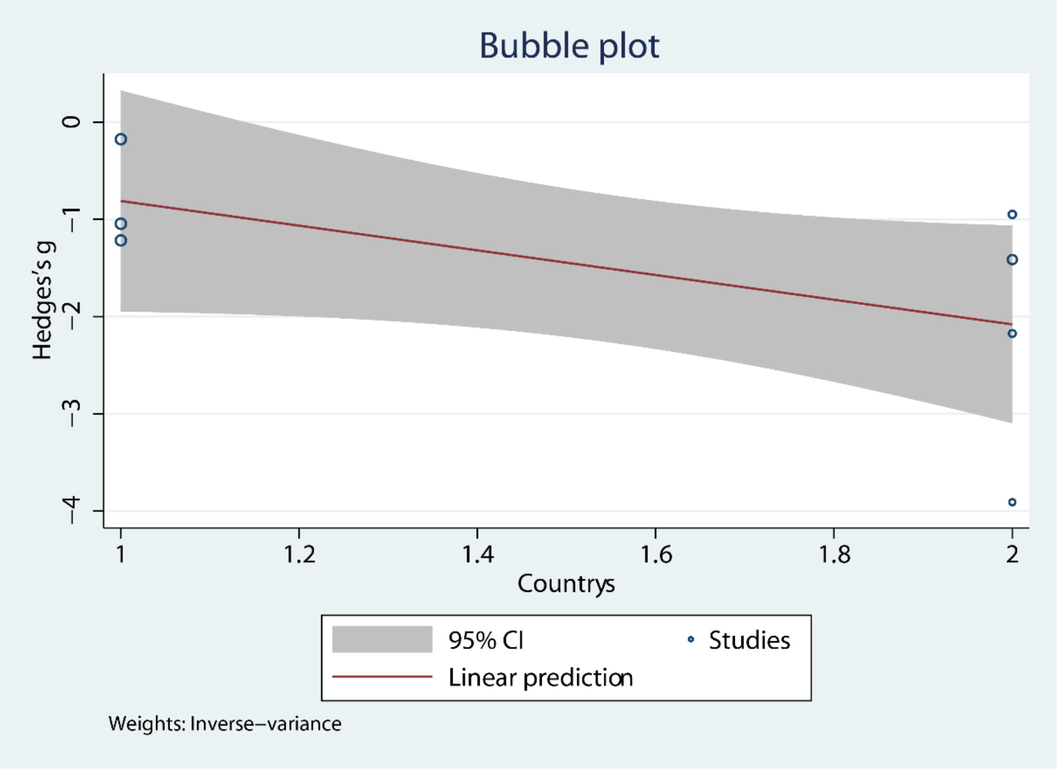

Fig S12 Sensitivity analysis of sleep quality in MHD patients.

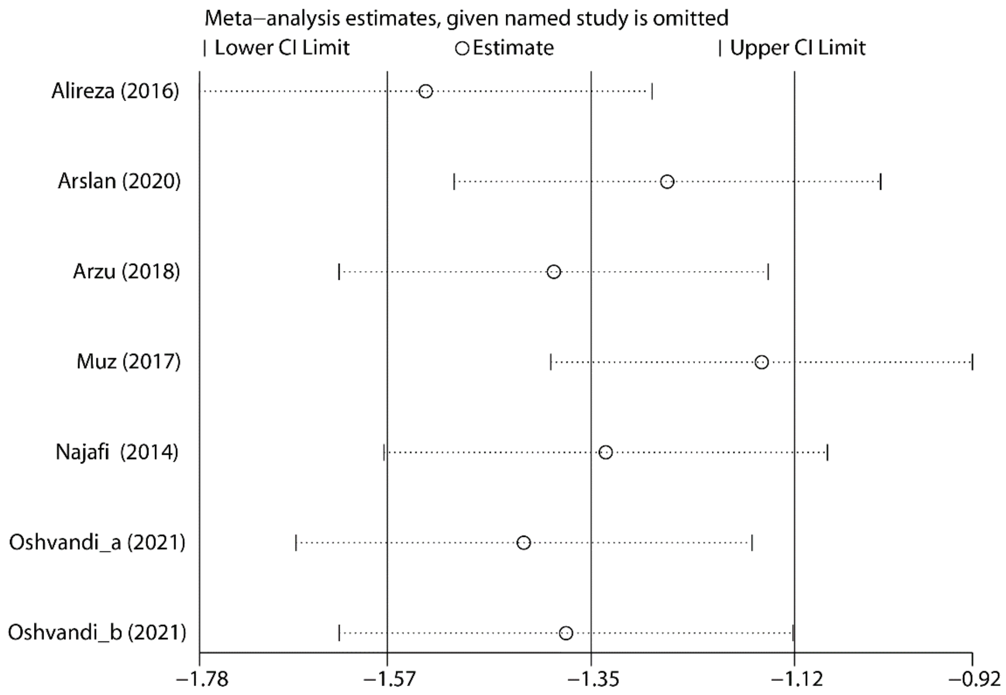

Fig S13 Subgroup analysis of arteriovenous fistula puncture pain in MHD patients.

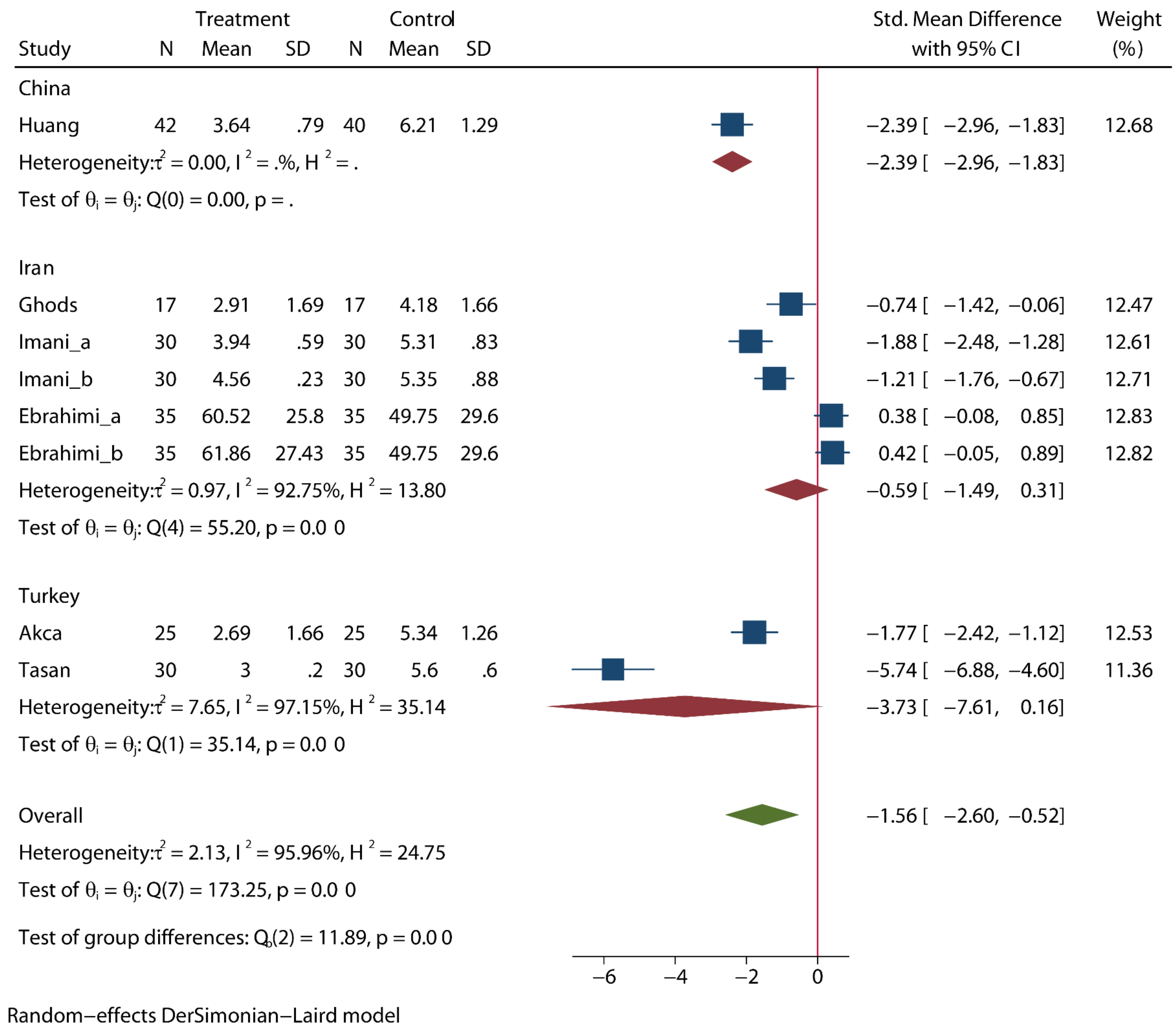

Fig S14 Meta-regression of publication year of arteriovenous fistula puncture pain in MHD patients.

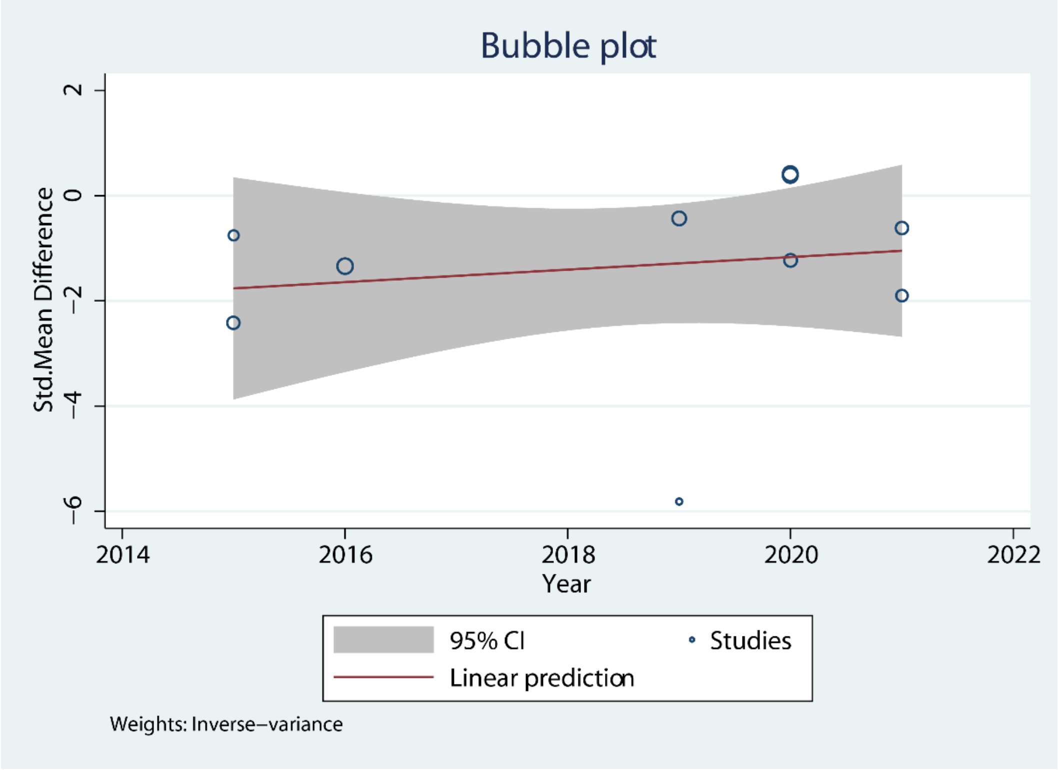

Fig S15 Meta-regression of study size of arteriovenous fistula puncture pain in MHD patients.

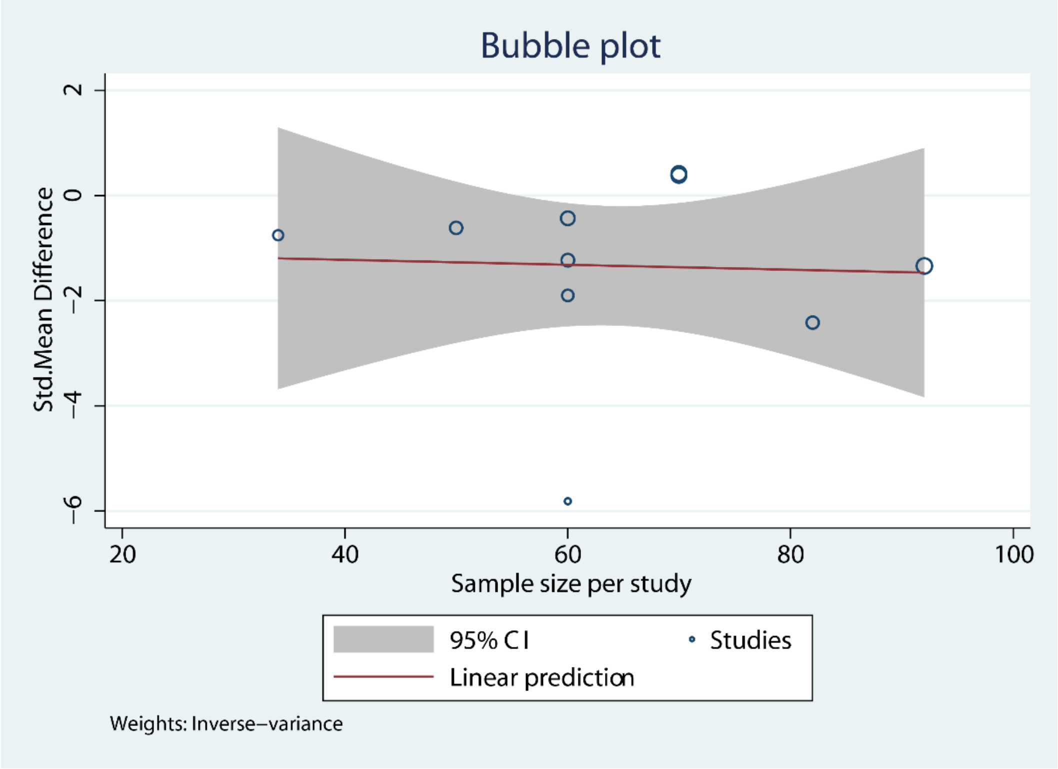

Fig S16 Meta-regression of publication country of arteriovenous fistula puncture pain in MHD patients.

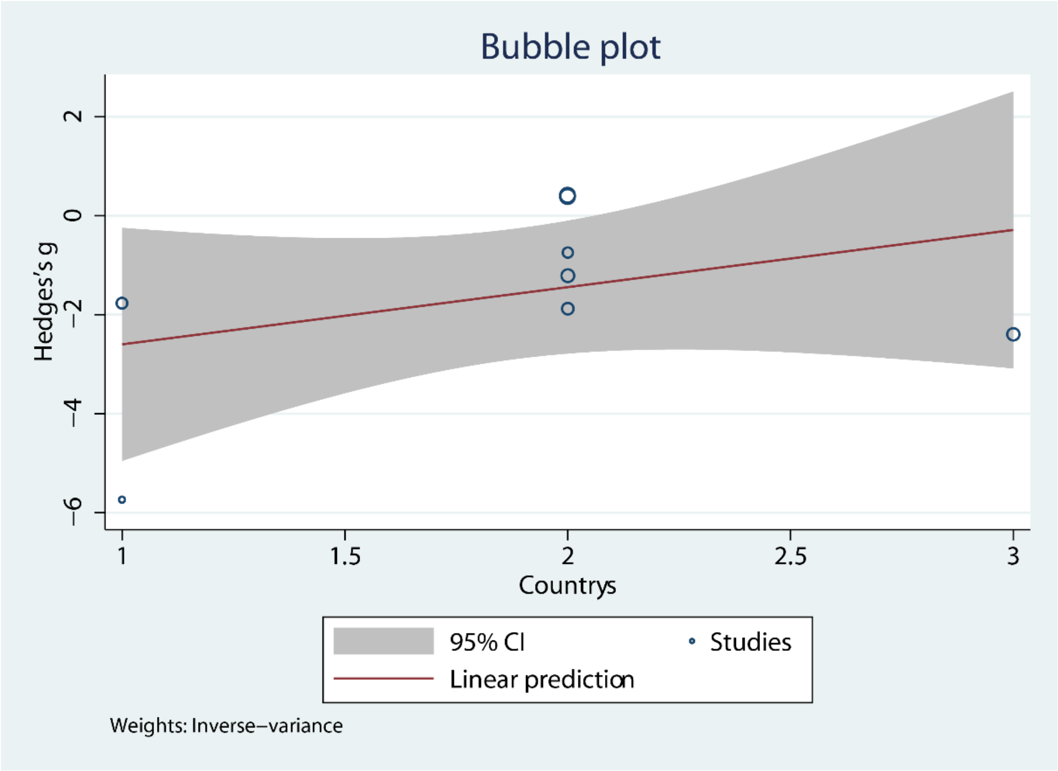

Fig S17 Sensitivity analysis of arteriovenous fistula puncture pain in MHD patients.

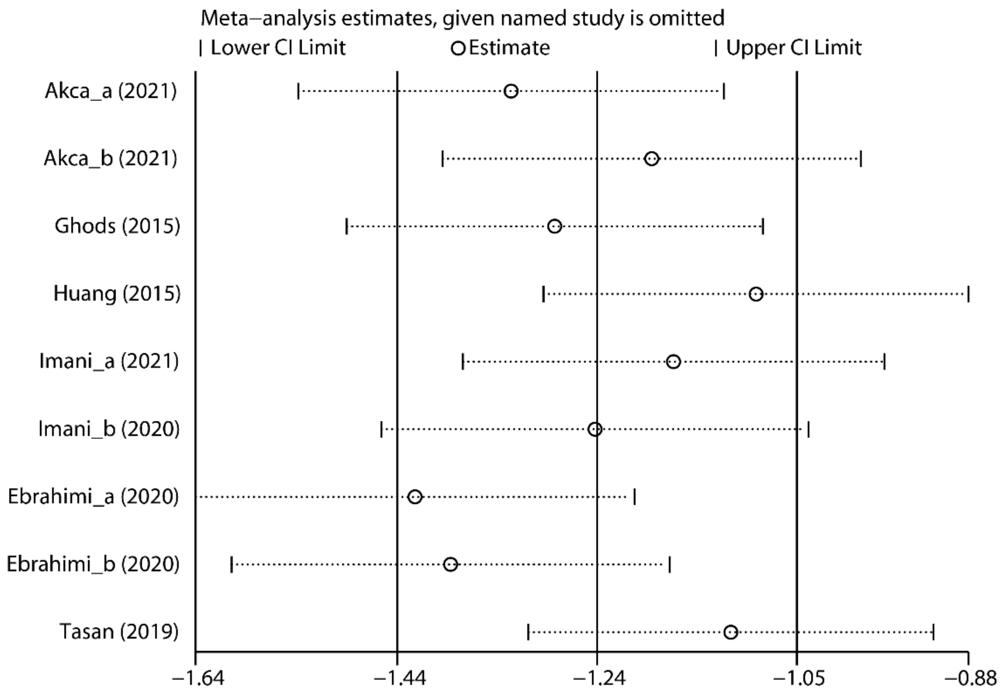

Information Classification: General

Fig S18 Subgroup analysis of anxiety in MHD patients.

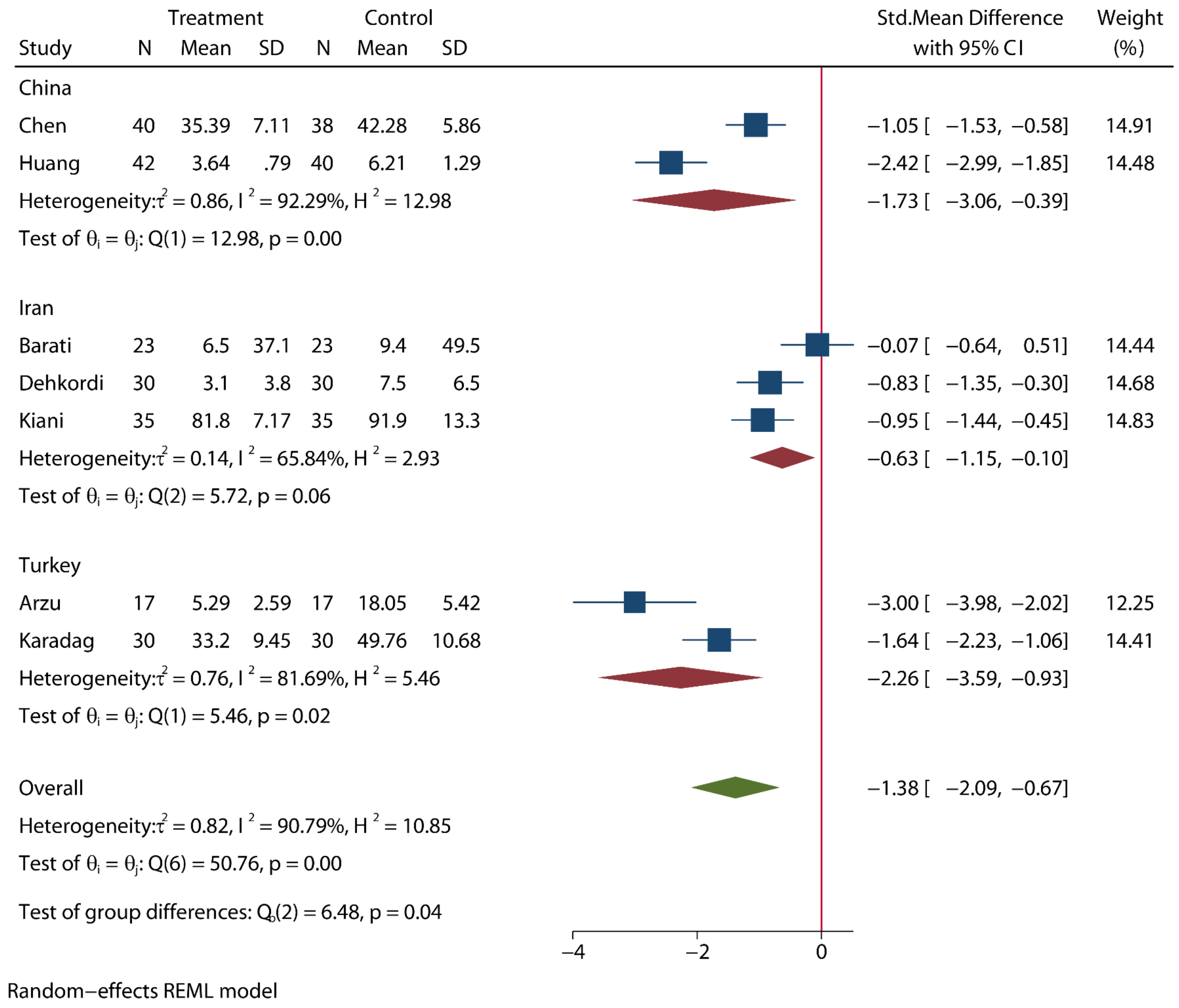

Fig S19 Meta-regression of publication year of anxiety in MHD patients.

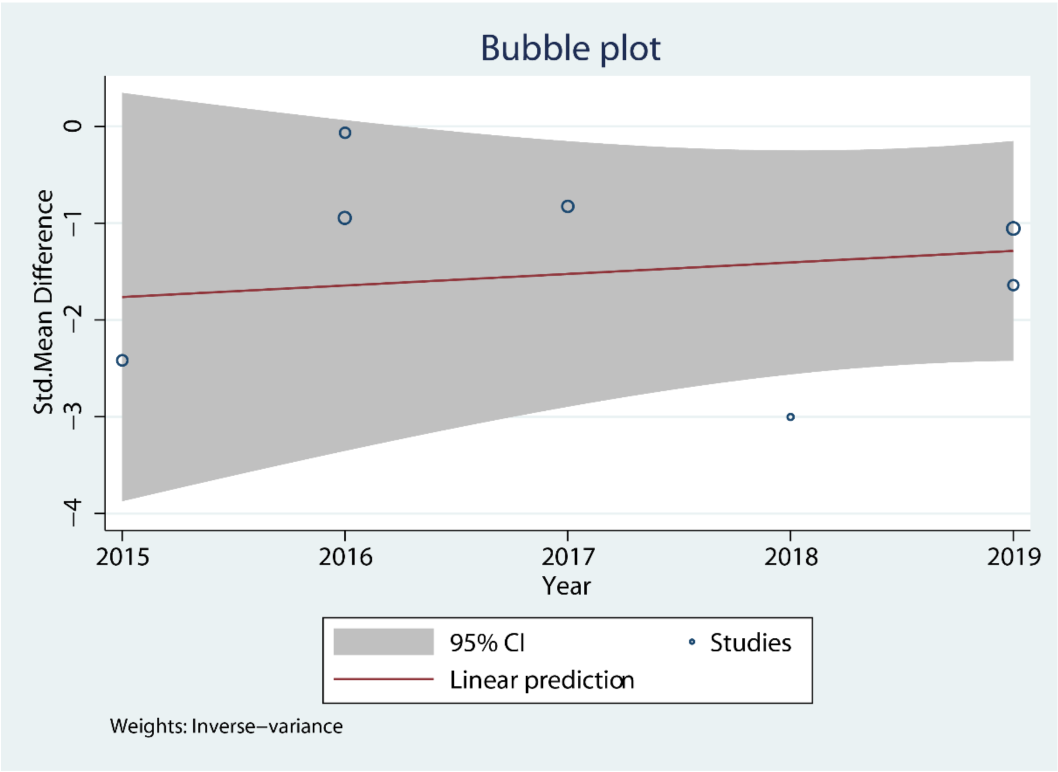

Fig S20 Meta-regression of study size of anxiety in MHD patients.

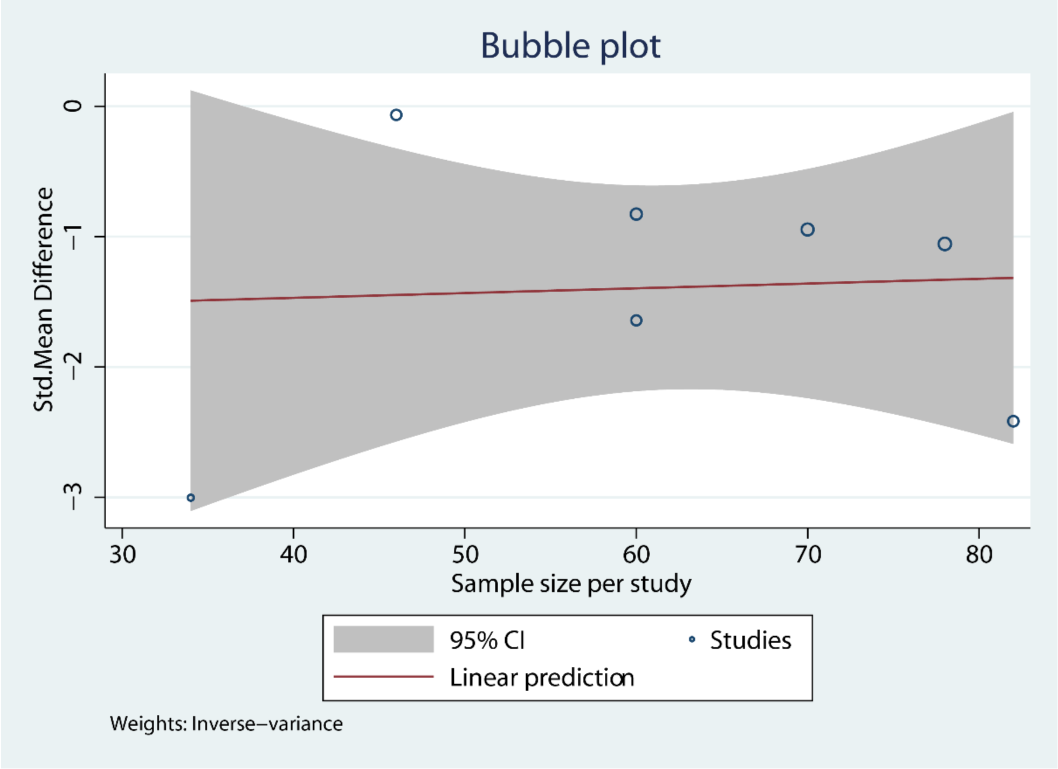

Fig.S21 Meta-regression of publication year of anxiety in MHD patients.

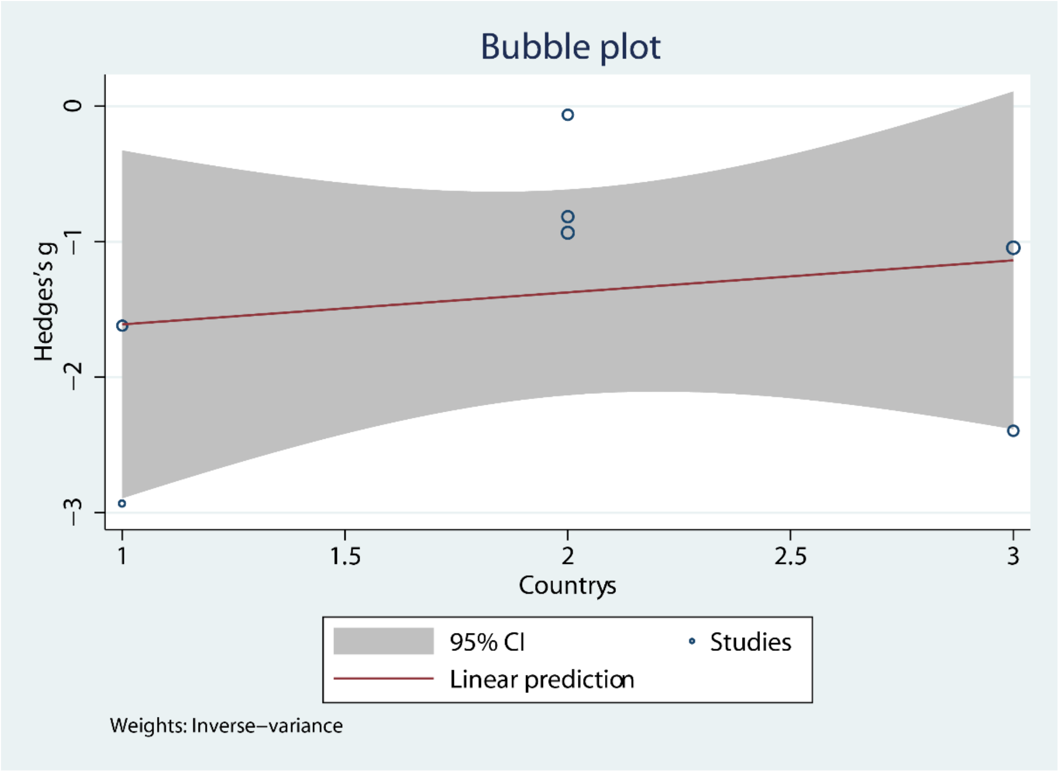

Fig S22 Sensitivity analysis of anxiety in MHD patients.

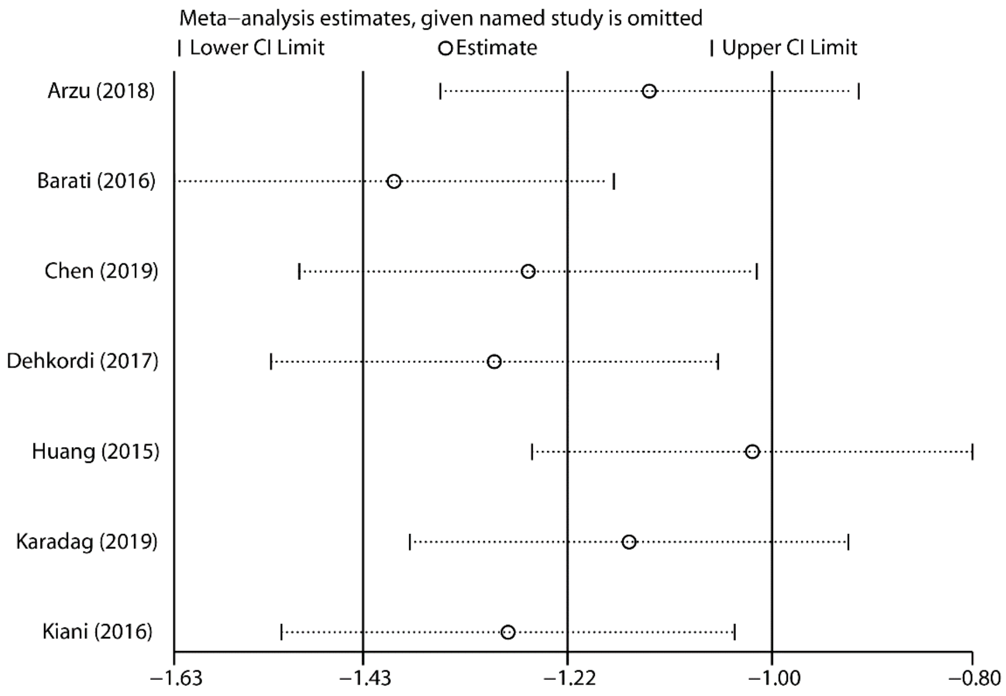

Supplement: Supplemental Material [file IRNF_A_2164202_SM4926.pdf]
